# A macroevolution-inspired approach to reveal novel antibiotic resistance mechanisms

**DOI:** 10.7554/eLife.101940

**Published:** 2026-06-08

**Authors:** Fernanda T Subtil, Teresa FG Machado, Holly Douglas, Joanna M Kirkpatrick, Mark Skehel, Acely Garza-Garcia, Luiz Pedro S de Carvalho

**Affiliations:** 1 Mycobacterial Metabolism and Antibiotic Research Laboratory, The Francis Crick Institute London United Kingdom; 2 Proteomics Scientific Technology Platform, The Francis Crick Institute London United Kingdom; 3 Department of Chemistry, The Herbert Wertheim UF Scripps Institute for Biomedical Innovation and Technology Jupiter United States; University of the Witwatersrand South Africa; University of the Witwatersrand South Africa

**Keywords:** macroevolution, mycobacterium species, antibiotic resistance, rifamycins, rifamycin ADP-ribosyltransferase, non-tubercular mycobacteria, Other

## Abstract

With the continuous rise in antibiotic resistance, novel methods that can reveal currently unknown antibiotic resistance mechanisms are essential to prepare and inform health responses and novel antibiotic discovery campaigns. Here, we built a library of species representative of the genus *Mycobacterium* and determined their antibiotic resistance profiles, allowing for the first time systematic multispecies comparisons. Analyzing antibiotic resistance in the context of other closely related yet diverse organisms revealed species with truly exceptional traits as well as general principles underpinning antibiotic resistance. Among these, we reveal that intrabacterial accumulation of antibiotics does not correlate with their potency at the species level. Our data also reveals that rifamycin resistance in mycobacteria is dominantly caused by antibiotic modification, contrary to what has been observed in *Mycobacterium tuberculosis*. Our data provides a solid starting point for the exploration of novel determinants of antibiotic resistance. We illustrate the utility of this species-level approach to discovery of novel traits by characterizing a previously unrecognized rifamycin-inactivating enzyme group that is present in a wide range of bacterial genera.

## Introduction

The discovery and successful clinical deployment of antibiotics is one of the most important breakthroughs in medical history, as it has dramatically reduced the morbidity and mortality in infections. Conversely, the extensive use of antibiotics has promoted the selection and propagation of mechanisms that enable bacteria to thrive regardless (WHO Team, 2015; Ventola, 2015). Not all resistance mechanisms can be attributed to human-derived antibiotic exposure, as many predate the clinical use of antibiotics and/or are observed in bacteria of no clinical importance (Cox and Wright, 2013). These mechanisms are derived from the natural evolution of species and the environment they occupy, as bacteria that co-inhabit niches with antibiotic-producing organisms naturally develop ways to avoid antibiotic-caused death (Dantas and Sommer, 2012; Finley et al., 2013). Of key importance, antibiotic resistance mechanisms do not have to evolve de novo, as they can be incorporated from other organisms via horizontal gene transfer (HGT).

Two main strategies are used to identify novel antibiotic resistance determinants. One clinical or host-biased approach ensues when a patient who is infected with a presumably antibiotic-sensitive species fails to improve upon treatment. Isolation and study of the resistant strain then leads to the identification of a novel determinant of antibiotic resistance. Several seminal discoveries of direct clinical importance have taken place in this manner (Andries et al., 2014; Farhat et al., 2019; Mathys et al., 2009). However, this approach mostly discovers incremental strain-specific mechanisms, such as single nucleotide polymorphisms (SNP) that alter antibiotic binding to its target. Another strategy involves ecological sampling and screening, using targeted approaches or metagenomics (D’Costa et al., 2011). The latter approach holds the promise to uncover truly novel mechanisms, although these mechanisms might never find their way into extant pathogens, and therefore will never represent a clinically relevant problem.

*Mycobacterium* is a genus that includes both environmentally and clinically relevant microorganisms. Mycobacteria can be found in most environments including rivers and lakes (Djouadi et al., 2016), soil (Hennessee et al., 2009), plant roots (Bouam et al., 2018), moss (Kazda et al., 1993), reptiles (Bergey, 1923), amphibians (Schwabacher, 1959), fish (Pourahmad et al., 2015), and mammals (Bojalil et al., 1962). A typical divide of the genus is based on growth rate in a defined solid medium when sub-cultured from highly dilute inoculum (Magee and Ward, 2012). A mycobacterial species is ‘fast-growing’ if it forms visible colonies within seven days; this phenotype is considered to be the ancestral state of the genus (Devulder et al., 2005). A species that takes longer than seven days to form mature colonies is classified as ‘slow-growing’, this category includes the most devastating disease-causing species *Mycobacterium ulcerans*, *Mycobacterium avium* complex (MAC), *Mycobacterium leprae*, and the *Mycobacterium tuberculosis* complex (MTBC). Until recently, human infections with mycobacteria other than the MTBC and *M. leprae*, that is non-tuberculous mycobacteria (NTM), were overshadowed by the TB burden but are now gaining increased attention due to their growing prevalence (Griffith et al., 2007). Some of the key NTM species that cause disease are the fast-growers *Mycobacterium abscessus* and *Mycobacterium fortuitum*, and the slow-growers *M. avium*, *Mycobacterium marinum*, *Mycobacterium xenopi*, *Mycobacterium gordonae,* and *Mycobacterium kansasii* (Johansen et al., 2020). NTM can infect a variety of tissues including the lungs, central nervous system, lymphatic system, joints, and skin (Johansen et al., 2020). As the frequency of NTM infections is increasing, so is the worry of their severity and resistance to treatment with available antibiotics. Contrasting with *M. tuberculosis* and *M. leprae*, which due to their isolation inside the host do not show HGT, NTM might acquire resistance determinants from environmental bacteria using phages, plasmids, and other mobile genetic elements (Abdulkadir et al., 2024; Zhang et al., 2022). Therefore, the potential of mycobacterial species to contain and disseminate antibiotic resistance determinants is likely as good as that of other environmental bacteria.

Here, we propose a new method to study antibiotic resistance by comparing antibiotic resistance profiles across related bacterial species (macroevolution), instead of comparing strains of the same species (microevolution) to identify previously unknown high-level antibiotic resistant species and understand general principles of antibiotic resistance in a genus. Then, taking advantage of the natural genetic diversity among species (largely encoded in their accessory genome) and their genetic similarity (encoded in their core genome), computational, molecular, and cellular approaches can more readily pinpoint resistance determinants. We demonstrate the power of this method by characterizing a previously unrecognized rifamycin-inactivating enzyme group that is widely distributed across bacterial genera.

## Results

### Building a diverse library of mycobacterial species

**Figure 1.**
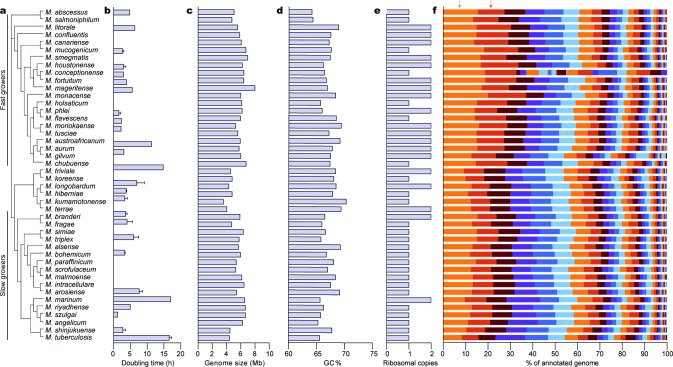
A diverse species library of the genus *Mycobacterium*. (**a**) Phylogenetic tree of mycobacterial species in our library calculated using the bcgTree pipeline v 1.1.0 [62, 65-68]. (**b**) Doubling time. (**c**) Genome size. (**d**) Guanine and cytosine percentage (GC%). (**e**) Ribosomal copies (rrn operon). (**f**) Gene ontology (GO) distribution. Colors from left to right represent the following GO categories: regulation of DNA-templated transcription (highlighted with a light orange arrow), transmembrane transport (highlighted with a dark orange arrow); amino acid, lipid, carbohydrate derivative, nucleobase-containing small molecule, and carbohydrate metabolic processes; generation of precursor metabolites and energy; sulfur compound, vitamin, and tRNA metabolic processes; DNA repair, protein modification process, signaling, cell wall organization or biogenesis, cellular modified amino acid metabolic process, DNA replication, DNA recombination, ribosome biogenesis, anatomical structure development, protein catabolic process, protein-containing complex assembly, protein maturation, nitrogen cycle metabolic process, intracellular protein transport, metal ion homeostasis, cell division, protein secretion, mRNA metabolic process, DNA integration, transport, organic substance transport, defense response to other organism, organic substance biosynthetic process, organic substance metabolic process, cellular process, nitrogen compound transport, regulation of gene expression, cellular biosynthetic process, and other metabolic processes.

A collection of 44 tractable mycobacterial species was assembled to cover most of the mycobacterial phylogenetic tree (Figure 1a). To evaluate the biological diversity of our library, we analyzed the ecological and genomic information available for the selected species. We determined doubling times in liquid culture in equivalent experimental conditions for 26 of the species (Figure 1c). The results revealed large differences within the genus. For example, *Mycobacterium szulgai* and *Mycobacterium flavescens* divided every 1.3 and 2.0 hr, respectively, while *M. marinum and M. tuberculosis* divided every 17.1 hr. Strikingly, among slow-growers a 13-fold change difference exists between the fastest and the slowest-growing species (*M. szulgai* and *M. tuberculosis*, respectively). The genome size of the library species is also highly different, with genomes as small as 3.59 Mbp and 4.08 Mbp for *Mycobacterium triviale* and *Mycobacterium koreense*, respectively, to genomes as large as 6.99 Mbp and 8.01 Mbp for *Mycobacterium smegmatis* and *Mycobacterium mageritense*, respectively (Figure 1d). These genome size differences suggest that the nature of the accessory genome varies widely from species to species. Interestingly, there is only a very small difference when comparing the average genome size of all fast and slow growers, 6.1 vs 5.8 Mbp, respectively. The high guanine-cytosine content (GC%) is a defining characteristic of mycobacteria. Within our library, GC% varies from 66.9% to 68.8% in slow growers and the *Mycobacterium terrae* clade respectively (Figure 1e). Next, we investigated the number of ribosomal RNA encoding genes (*rrn* operon), a feature that has often been linked to growth rate. Twenty-five species possess a single copy of the *rrn* operon while the other 19 possess two copies (Figure 1f). In general, but not always, ‘fast growers’ tend to have two copies while ‘slow growers’ one. In the genus *Bacillus* a correlation between growth rate and *rrn* operon copy number has been experimentally disproven (Valdivia-Anistro et al., 2015), and number of *rrn* operon copies could be related to response of resource availability (Klappenbach et al., 2000). Furthermore, comparative pangenome analyses conducted by Bachmann and collaborators suggested that growth limitation in slow growing mycobacteria might instead be related to loss of amino acid transporters (Bachmann et al., 2019). Finally, we analyzed the gene ontology (GO) distribution for our library in order to evaluate functional genetic diversity at the genome level. GO distribution varies noticeably across the analyzed genomes. The number of genes associated with transcription and membrane transport (categories indicated by arrows in Figure 1g) appeared to be particularly variable. We noted that much of the genome of several of these species is poorly annotated. Therefore, Figure 1 supports the potential usefulness of our library as a resource to investigate mycobacterial biology, antibiotic resistance, and pathogen evolution.

### Wide variation in antibiotic resistance in mycobacteria

**Figure 2.**
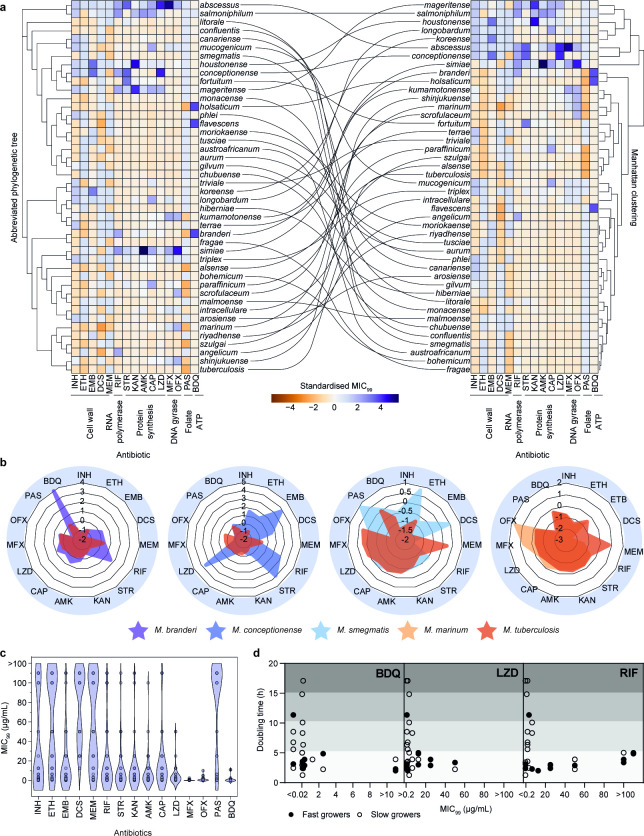
Antibiotic sensitivity mapping reveals complex patterns. (**a**) Heatmaps of overall MIC_99_ values. In the X axis, antibiotics are organized based on their mechanism of action; in the Y axis, mycobacterial species are organized phylogenetically in the left heatmap and based on their response to the set of antibiotics tested (Manhattan clustering) in the right heatmap. Colors represent the standardized MIC_99_ (mean/SD and centered scaled). Lower MIC_99_ values are in brown/orange and higher MIC_99_ values in lilac/purple. The details of the data can be found in Figure 2—figure supplement 2. Radar plots displaying the standardized MIC_99_ for all antibiotics tested. MIC_99_ ¬ values are normalized to be plotted in radar plots. All radar plots display the results for *M. tuberculosis* in orange. *Mycobacterium branderi* is displayed in purple, *Mycobacterium conceptionense* in dark blue, and *M. smegmatis* in light blue. (**c**) Violin plots showing the distribution of MIC_99_ values. In the X axis, the set of antibiotics tested; in the Y axis the MIC_99_ values in µg/mL. (**d**) Relationship between mycobacterial doubling time (X axis) and MIC99 for the antibiotics BDQ, LZD, and RIF (Y axis). Antibiotics targeting the cell wall are: isoniazid (INH), ethionamide (ETH), ethambutol (EMB), d-cycloserine (DCS), and meropenem (MEM); RNA/protein synthesis: rifampicin (RIF), streptomycin (STR), kanamycin (KAN), amikacin (AMK), capreomycin (CAP), and linezolid (LZD); DNA gyrase: moxifloxacin (MFX) and ofloxacin (OFX); folate metabolism: para-aminosalicylic acid (PAS); and ATP synthase: bedaquiline (BDQ). Figure 2—source data 1.Mass spectrometry data used to quantify rifampin in mycobacteria.

To harness the potential of our library, we tested the antibiotic potency and the extent of its variation across the *Mycobacterium* genus to identify biologically-relevant differences to be further studied. We determined minimal inhibitory concentrations (MIC_99_) for 15 antibiotics, spanning most of the classes employed to treat mycobacterial infections, including TB (Figure 2a, Supplementary file 1). We found that several species displayed at least one MIC_99_ value that is considerably different from the mean (Figure 2—figure supplements 1 and 2), highlighting the biological diversity of the genus with respect to antibiotic potency. As expected, the notoriously multi-drug resistant *M. abscessus* was resistant to several antibiotics (Figure 2a; Johansen et al., 2020; Luthra et al., 2018), yet *M. abscessus* was not the most resistant species studied. *M. mageritense*, *Mycobacterium salmoniphilum,* and *Mycobacterium houstonense* were highly resistant to most of the antibiotics tested. *M. abscessus* was somewhat sensitive to amikacin (AMK) and bedaquiline (BDQ), which is consistent with other findings in the literature (Jayasingam et al., 2017; Vesenbeckh et al., 2017). Also, the magnitude of the differences in MIC_99_ when compared to *M. tuberculosis* is remarkable, of the order of 100- to 1000-fold in some instances. Of note, when the data were re-ordered and unbiased clustered, based on the overall antibiotic sensitivity of each individual species, the species distribution was divided into three main clusters, which are dramatically different when compared to their phylogenetic positioning, as shown by the tanglegram between the two heatmaps in Figure 2a. To illustrate the absence of a taxonomic trend in antibiotic resistance, we explored in detail the clade composed of *Mycobacterium holsaticum*, *Mycobacterium phlei*, *M. flavescens*, *Mycobacterium tusciae,* and *Mycobacterium moriokaense* (Figure 2—figure supplement 3). While most antibiotics behaved similarly across this group (i.e. MIC_99_ FC <3 fold), *M. holsaticum* was highly sensitive to para-aminosalicylic acid (PAS) and highly resistant to BDQ. This higher sensitivity to PAS cannot be attributed to any known mechanisms. For example, the recently discovered mutations associated with lower methylene tetrahydrofolate reductase (Rv2172c) activity, leading to increased PAS sensitivity in *M. tuberculosis* (Yu et al., 2022), are not observed in the *M. holsaticum* homolog (WP_069405771.1, Figure 2—figure supplement 4). High-level BDQ resistance was also observed with *M. flavescens*. Interestingly, *M. flavescens* is unusually sensitive to d-cycloserine (DCS). The mechanisms underpinning these distinct responses to antibiotics are currently unknown. We also observed that once ordered by antibiotic sensitivity, *M. tuberculosis* is positioned at the middle of the heatmap and the number of NTM more resistant to antibiotics compared to *M. tuberculosis* is nearly equal to the number of NTM that are more sensitive. Based on the data above, obtained with 44 species, NTM are not intrinsically more drug resistant to the antibiotics tested than *M. tuberculosis*. In summary, antibiotic sensitivity varies dramatically across the *Mycobacterium* genus and our data provide the first quantitative blueprint of this variation.

**Figure 2—figure supplement 1.**
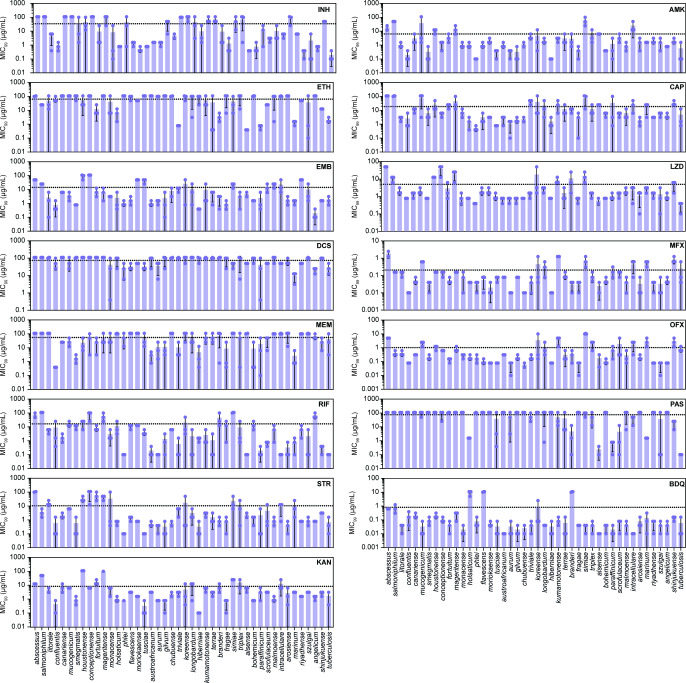
Detailed overall MIC_99_ quantifications against the mycobacterial species in our library. X axes represent the species tested in phylogenetic order, and Y axes are the MIC99 measurements in log_10_ scale. Dotted lines represent the mean MIC99 for each drug.

**Figure 2—figure supplement 2.**
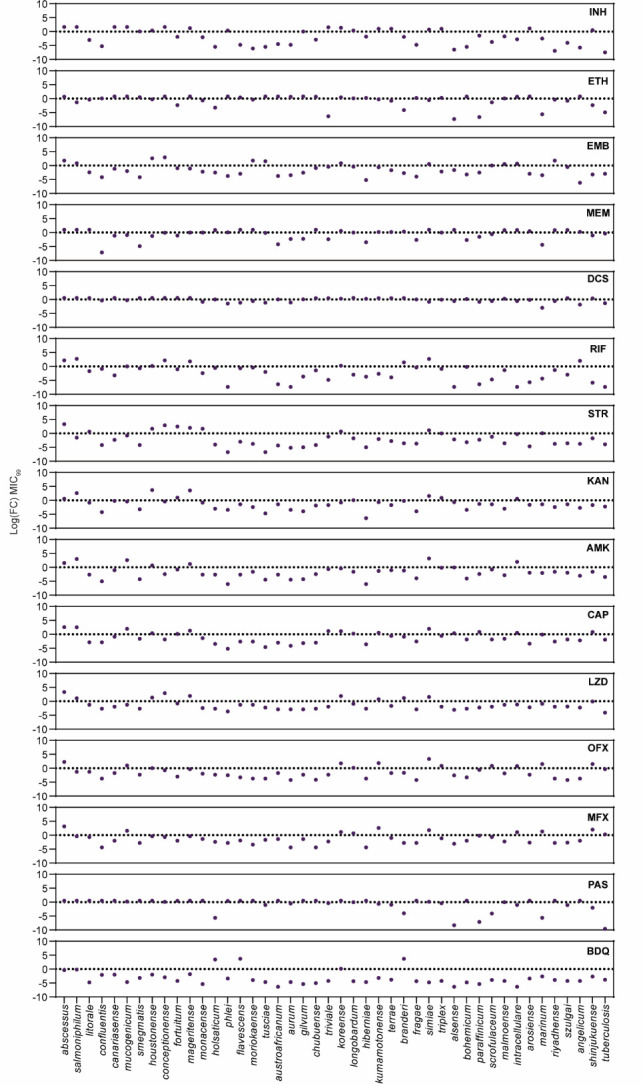
Log_2_(FC) MIC_99_ (Y axis) compared to the mean MIC_99_ for each antibiotic. Species organized by phylogeny (X axis). Dotted line represents the mean MIC_99_ for each antibiotic.

**Figure 2—figure supplement 3.**
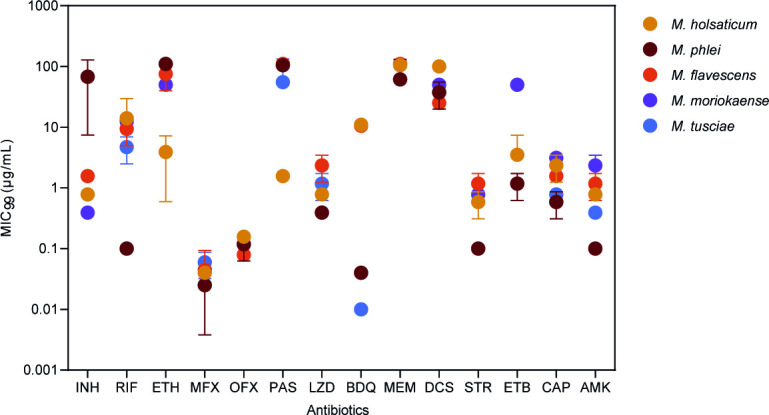
Detailed view of the *Mycobacterium holsaticum* group MIC_99_ values.

**Figure 2—figure supplement 4.**
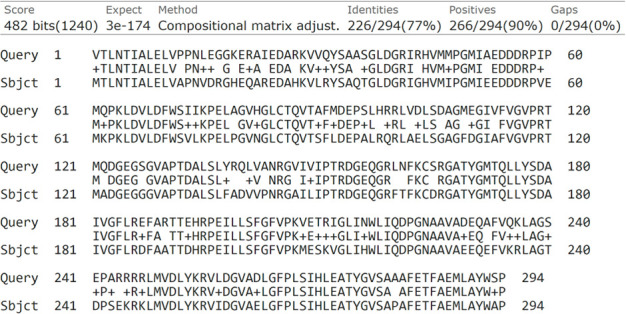
Primary sequence alignment of Rv2172c-encoded methylene tetrahydrofolate reductase and its homolog from *M. holsaticum*. Red arrows indicate residues that lead to decreased enzymatic activity and increased sensitivity to PAS.

There are striking differences in antibiotic response across the genus that highlight the value of a genus-wide approach to inform antibiotic research efforts. For example, *M. smegmatis* is frequently used as a model organism for *M. tuberculosis* in TB antibiotic discovery (Zampieri et al., 2018), but it displays a completely different sensitivity profile from *M. tuberculosis*, being highly resistant to PAS, ethionamide (ETH), DCS, and RIF. Conversely, our results suggest that *M. marinum* is a better *M. tuberculosis* proxy (Takaki et al., 2012) as both have a similar sensitivity profile, except to ofloxacin (OFX; Figure 2b). The overall distribution of antibiotic potency (MIC_99_) against different species is shown in Figure 2c. Cell-envelope-targeting antibiotics and PAS exhibit a weaker potency across the genus, while antibiotics that target protein synthesis, DNA gyrase and the ATP synthase on average displayed an overall lower MIC_99_, indicating that most mycobacteria are sensitive to them. From the antibiotics that inhibit protein synthesis, linezolid (LZD) displays the lowest overall MIC_99_ and was effective against most species (Figure 2c). To verify whether there is a correlation between doubling time and sensitivity to antibiotics, we compared the doubling time of a subset of species (Figure 1c) with the MIC_99_ of a subset of antibiotics. No correlation is apparent between growth rate and antibiotic sensitivity in mycobacteria (Figure 2d). Given the antibiotic response profiles observed, we selected BDQ, LZD, and RIF to explore the molecular causes of these dramatic changes in antibiotic potency observed across the *Mycobacterium* genus.

### Intra-bacterial antibiotic accumulation does not predict potency

**Figure 3.**
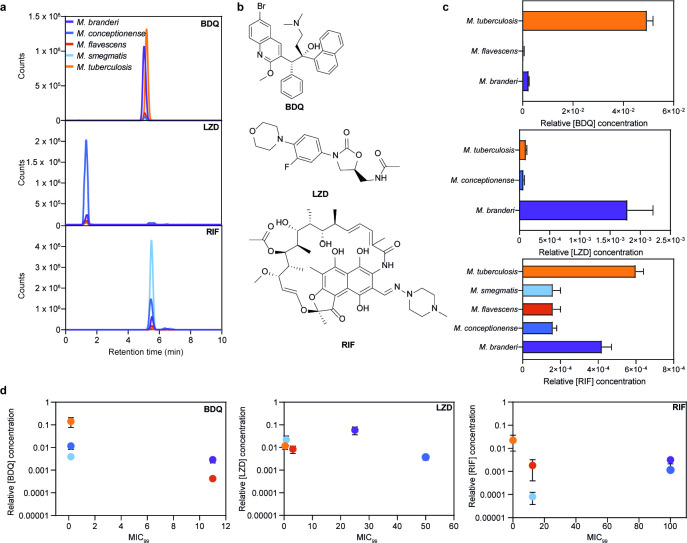
Intrabacterial antibiotic concentration does not correlate with potency. (**a**) Positive mode extracted ion chromatograms (EICs) of whole-cell extracts of mycobacteria treated with selected antibiotics. BDQ (m/z 555.1642), LZD (m/z 338.1511), and RIF (m/z 823.4124). (**b**) Chemical structure of BDQ, LZD, and RIF. (**c**) Relative intracellular antibiotic concentration, obtained by comparing the peak height of the samples with and injection of 10 µM of antibiotic, then normalized by the concentration of antibiotic used to treat the cells. (**d**) Relative intracellular antibiotic concentrations in relation to the MIC_99_. Data in (c) and (d) come from independent experiments.

We employed liquid chromatography–time-of-flight mass spectrometry (LC-MS) to determine the relative intrabacterial (IB) concentration of antibiotic ([ABX]_IB_) with an antibiotic concentration in the growth medium of 6×MIC_99_. Generally, [ABX]_IB_ is a function of three parameters: antibiotic uptake, efflux, and modification. Figure 3a shows extracted ion chromatograms (EICs) in five mycobacterial species for BDQ, LZD, and RIF (Figure 3b). Quantification of [BDQ]_IB_, [LZD]_IB_, and [RIF]_IB_ illustrates the variability and the magnitude of the changes observed in [ABX]_IB_, spanning from 2- to 200-fold (Figure 3c). As the experiment was performed at a concentration of antibiotic proportional to its MIC_99_, and therefore antibiotics were equally potent, we replotted these data as a function of each antibiotic MIC_99_ (Figure 3d). Only for BDQ we could observe some correlation between antibiotic potency and [BDQ]_IB_ which could be indicative of efflux playing a role in antibiotic efficacy. In the case of RIF, where no correlation between antibiotic potency and its accumulation in mycobacteria is observed (Figure 3d), factors other than uptake and efflux must be at play.

#### A minor role for pre-existing target modification in RIF resistance

**Figure 4.**
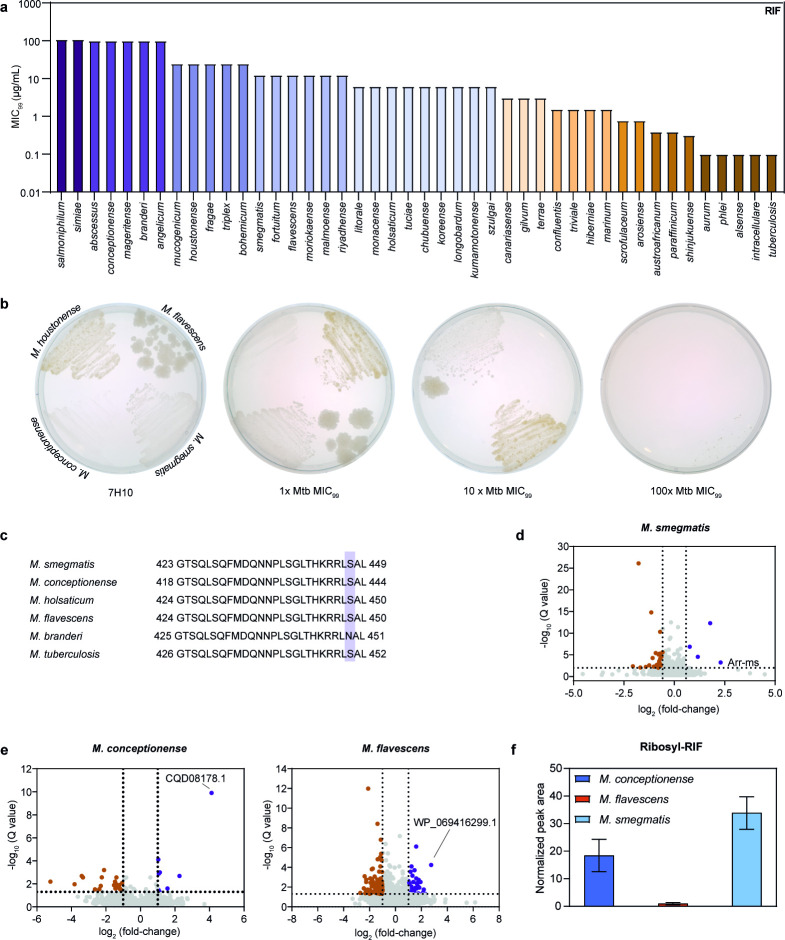
High-level rifampicin resistance is caused by rifamycin modification in selected mycobacteria. (**a**) RIF MIC_99_ values for the mycobacterial species in our library organized in decreasing MIC_99_ value order. (**b**) Cultures of selected species on solid medium (7H10) containing RIF at different concentrations, starting at 1× *M. tuberculosis* MIC. (**c**) Comparison of the amino acid residue sequence of the rifampicin resistance-determining region (RRDR) of RpoB in selected mycobacterial species; the only residue that differs is Ser 450 in *M. branderi*. (**d and e**) Volcano plots showing the differential protein expression in whole-cells with and without RIF revealing inducible expression of RIF ADP-ribosyltransferase 1 (Arr-1) in *M. smegmatis*, *M. conceptionense,* and *M. flavescens*. (**f**) Detection and quantification of ribosyl-RIF in whole-cell extracts by LC-MS.

Considering the importance of rifamycins for the treatment of TB, leprosy, Buruli ulcer, MAC, and *M. kansasii* infections, we focused on RIF resistance mechanisms operating in mycobacteria. Figure 4a highlights the diversity in RIF potency across our library, ranging from an MIC_99_ of more than 100 µg/mL to less than 0.2 µg/mL. Arranging species by decreasing MIC_99_ value highlights that there are species better suited for the identification of target-mediated resistance mechanisms (dark orange), and species that are better suited for the identification of non-target-based resistance mechanisms (dark purple). At this stage, we focused our work on four species, all of which are resistant (MIC_99_=12.5 µg/mL) or super-resistant (MIC_99_=100.0 µg/mL) to RIF, compared to *M. tuberculosis* (MIC_99_=0.9 µg/mL): *M. smegmatis* and *M. flavescens* (MIC_99_=12.5 µg/mL), *M. houstonense* (MIC_99_=25.0 µg/mL), and *Mycobacterium conceptionense* (MIC_99_=100.0 µg/mL; Figure 4b).

**Figure 4—figure supplement 1.**
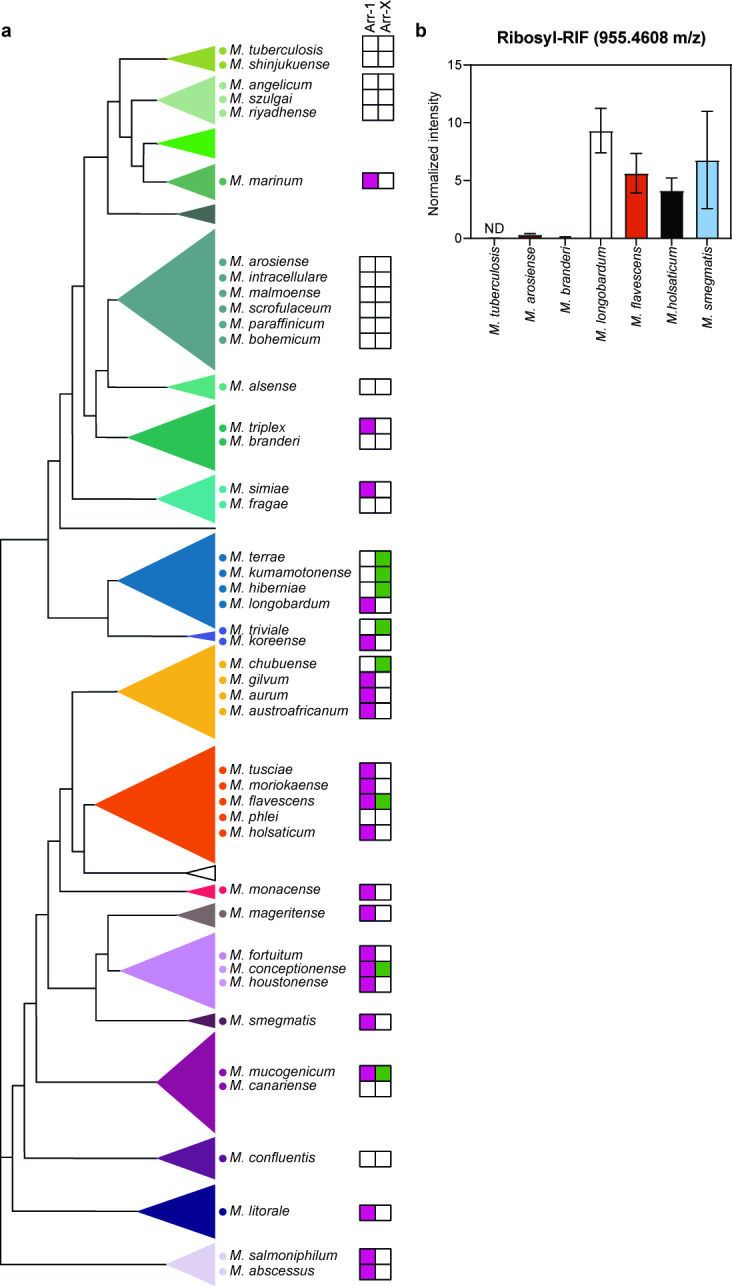
Phylogenetic distribution of RIF ADP-ribosyl transferases present in the mycobacterial species in our library. (**b**) Quantification of ribosyl-RIF in some mycobacterial species that encode Arr-1 and/or Arr-X. Not detected (ND).

In *M. tuberculosis*, the fixation of mutations that decrease the affinity of RIF to the RNA polymerase β subunit (RpoB) represents the dominant cause of RIF resistance (Farhat et al., 2019; Lawn and Nicol, 2011), and therefore target modification is an obvious starting point to explore probable mechanisms of resistance to the rifamycin class of antibiotics in mycobacteria. Figure 4c shows the rifampicin resistance determining region (RRDR), the segment of RpoB where most mutations conferring resistance to RIF are found. Except for *Mycobacterium branderi*, no amino acid variations are found in our species of interest. The rifampicin (RIF) binding pocket is generally conserved, but *M. branderi* has an S450N mutation in the RRDR region. While this specific mutation has not been found in clinical isolates (Farhat et al., 2019), it is located at the rifamycin binding site and may therefore confer resistance. While S450N mutations have not yet been observed in *M. tuberculosis*-related mutations like S450Q have been linked to resistance. In contrast, *M. conceptionense*, *M. flavescens*, and *M. smegmatis* show no target sequence differences that explain their resistance. This observation suggests that in contrast to *M. tuberculosis*, most mycobacteria are not resistant to RIF due to variations in the RIF binding region of RpoB.

As (RIF)_IB_ or RpoB variation cannot account for the observed resistance to RIF, we evaluated the remaining major mechanism of resistance to rifamycins, drug modification. RIF modification is widely found in nature and is carried out by various enzyme types, including phosphotransferases, glycosyltransferases, ADP-ribosyltransferases (ARTs), and monooxygenases (Spanogiannopoulos et al., 2014; Spanogiannopoulos et al., 2012; Koteva et al., 2018; Baysarowich et al., 2008; Imai et al., 1999; Dabbs et al., 1995). Importantly, a RIF-ART homolog has been characterized in *M. smegmatis* (Imai et al., 1999); it is encoded by the gene MSMEG_1221, also known as *arr*-ms. The arr-ms encoded RIF ADP-ribosyltransferase (RIF-ART) has been shown to be the sole determinant of RIF resistance in *M. smegmatis* by chemical and genetic methods (Zheng and Lupoli, 2021; Ganapathy et al., 2023). We employed proteomics to first check whether Arr-ms is expressed in the absence of RIF and if it is differentially expressed in the presence of RIF. Figure 4d shows that expression of Arr-ms is stimulated (5.6-fold) in the presence of RIF at 6×MIC_99_, and therefore, proteomics can assist on the identification of RIF modifying enzymes in mycobacteria. Next, we evaluated whether the annotated Arr homologous proteins in *M. conceptionense* (SAMEA3305051) and in *M. flavescens* (SAMN05729960) were also induced in the presence of RIF (Figure 4e), this was indeed the case (4.12- and 2.75-fold change, respectively). To confirm that these putative RIF-ARTs are inactivating RIF, we employed LC-MS to identify ribosyl-RIF (m/z 955.4601), a fragment of the larger ADP-ribosyl-RIF product, which fragments under our LC-MS conditions. Figure 4f illustrates that ribosyl-RIF was observed in *M. smegmatis*, *M. conceptionense,* and *M. flavescens* treated with RIF. Additionally, other mycobacteria with annotated putative *arr* genes also displayed high levels of RIF ADP-ribose (Figure 4—figure supplement 1), indicating that RIF modification, and precisely ADP-ribosylation, is the dominant mechanism of resistance to RIF in mycobacteria.

### A novel group of rifamycin ADP-ribosyltransferases

**Figure 5.**
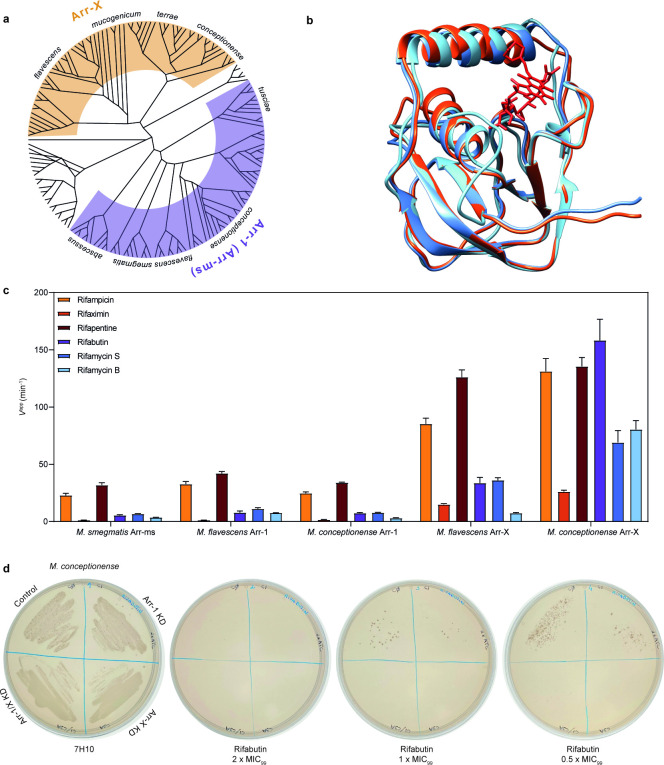
Characterization of a novel rifabutin-ADP ribosyltransferase in mycobacteria. (**a**) Phylogenetic tree of mycobacterial RIF ADP-ribosyltransferases (RIF-ARTs) and related PFAM family PF12120 sequences. Many mycobacterial species encode the equivalent of *M. smegmatis* RIF-ART (MSMEG_1221; Arr-1/ms in purple), and some mycobacterial species encode a previously unidentified sister group we have named Arr-X (dark orange). See Supplementary file 5 for detailed information of the sequences in the tree. (**b**) Ribbon representation of the crystal structure of *M. smegmatis* RIF-ART (PDB code 2HW2; light blue) with RIF bound (red) overlaid with the AlphaFold2 models of *M. conceptionense* Arr-X (dark blue) and *M. flavescens* Arr-X (dark orange) (Jumper et al., 2021). (**c**) Apparent velocity of reaction of each of the enzymes (X axis) with different rifamycins as substrate. (**d**) *M. conceptionense* single and double knockdown (KD) arr strains in the presence of rifabutin.

In order to have a comprehensive understanding of the distribution of Arrs in mycobacteria, we mined for *arr* sequences in reference genomes and built a phylogenetic tree. Arr proteins were found to be widespread in both fast- and slow-growing mycobacteria, but in a dispersed pattern suggesting that both local vertical inheritance and gene losses and acquisitions have taken place. Surprisingly, mycobacterial Arrs form two orthologous groups (Figure 5a; Supplementary file 2). One of the groups, previously known, which we designated Arr-1, corresponds to sequences closely related to Arr-ms (median sequence identity of 80%). Arr-1 group members are predominantly Actinomycetota of the orders Geodermatophilales, Propionibacteriales, Micrococcales, and Mycobacteriales. The second group, discovered here, which we have named Arr-X, is taxonomically more broadly distributed, including members from Actinomycetota, Bacillota, Pseudomonadota, and Bacteroidota. Within mycobacteria, more species have an *arr*-1 gene than *arr*-X and interestingly, a few species have both, for example *M. conceptionense* and *M. flavescens* (Supplementary file 5). *M. conceptionense* Arr-1 (Uniprot A0A0U1D6J3) and Arr-X (Uniprot A0A0U1DL14) share 50% identity and 63% similarity (BLOSUM62). The equivalent of the three residues showed by Baysarowich and collaborators to be necessary for enzymatic activity in Arr-ms (Asp84, His19, and Tyr49) are conserved in all mycobacterial Arr-1 and Arr-Xs, suggesting that they are all active ADP ribosyltransferases (Baysarowich et al., 2008). However, the hydrophobic nature of the RIF binding cleft of Arr-ms is not completely preserved in the Arr-X group (Figure 5b; Supplementary file 3) hinting at probable differences in substrate binding preference.

To assess that Arr-X enzymes are indeed rifamycin-ARTs and to understand why some species have two *arr* genes, we cloned, overexpressed, purified, and tested the enzymatic activity of Arr-ms (as a control) and Arr-1 and Arr-X from both *M. conceptionense* and *M. flavescens*. Figure 5c displays the catalytic activity (*V*^app^, as a surrogate of the specificity constant *V*/*K*) of the different proteins with six rifamycins. All Arr-1 enzymes had similar activity and substrate preference, but Arr-X enzymes were much superior at inactivating rifamycins. For example, *M. flavescens* Arr-X is four-fold faster with rifapentine than Arr-ms. Surprisingly, *M. conceptionense* Arr-X is 30-fold faster to inactivate rifabutin, compared to Arr-ms. These results therefore demonstrate that Arr-Xs are not only *bona fide* rifamycin inactivating enzymes, but also that they are significantly more efficient than Arr-1s. We also determined the MIC_99_ for different rifamycins in selected species (Supplementary file 4). Interestingly, these species are considerably resistant to all rifamycins except for rifabutin, for which the MIC is at a lower concentration. To probe whether Arr-X is active *in bacterio*, we used CRISPR interference to reduce the transcription of *arr*-1, *arr*-X and both genes in *M. conceptionense. M. conceptionense* continued to be resistant to rifabutin upon *arr*-1 silencing but became more sensitive when *arr*-X was knocked down (Figure 5d). Thus, Arr-X is an active ‘rifabutinase’ that confers rifamycin resistance in *M. conceptionense*.

## Discussion

Discovery of unknown mechanisms for high-level antibiotic resistance in nature is essential for efficient antibiotic discovery and development and for the continuous treatment of patients. Our ability to discover and improve antibiotics making them bypassing and evading resistance mechanisms is illustrated by the discovery of clavulanic acid and penems, and the rational development of glycylcyclines and more recently ETH booster compounds, which completely sensitize ETH-resistant *M. tuberculosis* to ETH (Gutierrez et al., 2005, Willand et al., 2009). Here, we propose a powerful approach for the discovery of novel antibiotic resistance determinants. Our strategy consists of mapping and comparing the antibiotic response profile of a library of tractable species representative of the entire genus *Mycobacterium* (macroevolution) instead of strains of the same species (microevolution). This approach is applicable for the study of many biological traits and to other bacterial genera.

Using our mycobacterial library, we identified for the first time high- and ultra-high-level intrinsic resistance (Cox and Wright, 2013) to many of the antibiotics tested. Of note, the identified resistant phenotypes are naturally occurring and not a result of mutations due to exposure to the antibiotic in the clinic – which is the more traditional approach for probing mechanisms of antibiotic resistance. Our observations revealed that resistance profiles are highly variable across the genus and do not follow phylogeny, implicating HGT as the key mechanism for acquisition of resistance determinants and evolution of antibiotic resistance in mycobacteria (Durão et al., 2018). While *M. tuberculosis* is famously regarded as ‘genetically locked’, which has not carried out HGT since it arose and underwent clonal expansion 35,000 years ago (Ankenbrand and Keller, 2016), the majority of species in the genus clearly exchanges DNA via plasmids, phages and other means. Our study revealed that resistance levels to BDQ, LZD, and RIF were particularly divergent across the genus and often could not be explained by our current knowledge of antibiotic resistance mechanisms (Andries et al., 2014; Farhat et al., 2019; Zampieri et al., 2018). We found that resistance to these antibiotics in mycobacteria does not correlate with uptake/efflux mechanisms in the species tested and it does not correlate with growth rate. Identification of mycobacterial species highly resistant to BDQ and LZD is worrisome as most of these species, if not all, have never been exposed to these drugs.

We illustrated the power of this comparative method by characterizing a previously unrecognized group of rifamycin-inactivating enzymes (Arr-X) that is present in a wide range of bacteria including Actinomycetes, Bacilli, and Gammaproteobacteria. We found that several mycobacterial species have the gene coding for the RIF inactivating enzyme RIF-ART (Arr-1), the founding member of this group of enzymes (Baysarowich et al., 2008) and revealed that some species also code for a homologous protein Arr-X. Arr-X enzymes are not only faster RIF-ARTs but also display higher specificity to rifabutin. The existence of a superior rifabutin-inactivating enzyme in several mycobacterial species might jeopardize the use of rifabutin, currently an antibiotic of choice to treat *M. avium* complex-caused infections and other infections. Novel inhibitors of these two distinct Arr enzymes (Zheng and Lupoli, 2021; Ganapathy et al., 2023) might become essential to re-sensitize mycobacteria against rifamycins and against rifabutin.

## Materials and methods

### Mycobacterial species and cultures

Mycobacterial species were acquired from the German Collection of Microorganisms and Cell Cultures GmbH – DSMZ (Braunschweig, Germany): *M. abscessus*, 44196; *M. salmoniphilum*, 43276; *Mycobacterium litorale*, 45785; *Mycobacterium confluentes*, 44017; *Mycobacterium canariense*, 44828; *Mycobacterium mucogenicum*, 44124; *M. houstonense*, 44676; *M. conceptionense*, 45102; *M. fortuitum*, 46621; *M. mageritense*, 44476; *Mycobacterium monacense*, 44395; *M. holsaticum*, 44478; *M. phlei*, 43239; *M. flavescens*, 43991; *M. moriokaense*, 44221; *M. tusciae*, 44338; *Mycobacterium austroafricanum*, 44191; *Mycobacterium aurum*, 43999; *Mycobacterium gilvum*, 45189; *Mycobacterium chubuense*, 44219; *M. triviale*, 44153; *M. koreense*, 45576; *Mycobacterium longobardum*, 45394; *Mycobacterium hiberniae*, 44241; *Mycobacterium kumamotonense*, 45093; *Mycobacterium terrae*, 43227; *M. branderi*, 44624; *Mycobacterium fragae*, 45731; *Mycobacterium simiae*, 44165; *Mycobacterium triplex*, 44626; *Mycobacterium alsense*, 45230; *Mycobacterium bohemicum*, 44277; *Mycobacterium paraffinicum*, 44181; *Mycobacterium scrofulaceum*, 43992; *Mycobacterium malmoense*, 44163; *Mycobacterium intracellulare*, 43223; *Mycobacterium arosiense*, 45069; *M. marinum*, 44344; *Mycobacterium riyadhense*, 45176; *M. szulgai*, 44166; *Mycobacterium angelicum*, 45057; *Mycobacterium shinjukuense*, 45663; *M. tuberculosis* H37Rv, lab collection; *M. tuberculosis* H37Ra, lab collection; and *M. smegmatis* mc^2^ 155, from ATCC, 700084.

The species comprised in our library were selected based on (i) broad genus coverage; (ii) diversity with respect to niche/pathogenicity; (iii) availability of genome sequence; (iv) availability of the type or laboratory strain; and (v) ability to grow on Middlebrook 7H9 culture medium. Upon arrival, long-term (–80 °C), short-term (–20 °C), and agar plate stocks were prepared according to DSMZ’s protocols.

### Mycobacterial diversity

Genotypic and phenotypic diversity is an essential feature required for our approach. Genome size and GC% values were obtained from NCBI Microbial Genomes (Durão et al., 2018) and ribosomal copy number was annotated using rrnDB (Stamatakis, 2014) or BLAST. When more than one hit was obtained, the genomic sequence and context were analyzed to confirm duplication. The growth rate of selected species was determined by turbidity measurements (OD_600_) taken in equal intervals. Species were selected based on experiments carried out throughout the study (e.g. correlating growth rate with antibiotic levels or requiring growth rate data for biomass matching in metabolomics experiments). We inoculated 100 mL Middlebrook 7H9 broth complete in roller bottles. Middlebrook 7H9 broth complete contained 10% Albumin-Dextrose-Catalase (ADC) supplement, 0.05% Glycerol, and 0.05% Tyloxapol. For all species, cultures were incubated at 37 °C, except for *M. marinum*, *M. abscessus*, *M. angelicum*, *M. conceptionense*, *Mycobacterium fallax*, *M. salmoniphilum,* and *M. tusciae* which were grown at 30 °C. Growth rate was calculated using the specific growth rate formula (Stoddard et al., 2015). Finally, encoded genes were classified based on their gene ontology using OmicsBox’s functional analysis tool, with default parameters (Maier et al., 2000; OmicsBox, 2019).

### Minimal inhibitory concentration measurements

Our mid-throughput minimal inhibitory concentration assay (MIC_99_) was adapted from previously described protocols (Götz et al., 2008). Briefly, in sterile Eppendorf tubes, each antibiotic was diluted in the recommended solvent to 4 mg/mL. Fluoroquinolones and BDQ were diluted to 0.4 mg/mL. Subsequently, 200 µL of each drug was added to column 10 of a 96-well plate. In the same plate, 100 µL of DMSO or water were added to columns 1–9 and 11. The drug titration was performed using a multi-channel pipette, using the volume 100 µL for 1:2 dilutions. The remaining 100 µL left over from column 1 was added to column 12, which is the negative control (contamination control). A secondary replicate plate was prepared in the same manner, and then combined for a final volume of 200 µL in each well. These plates were the master plates which were then copied into 36 new plates by transferring 5 µL of each well using Biomek FXᴾ Liquid Handling Automation (Beckman Coulter, California, USA). Once antibiotics were in the wells, 195 µL of Middlebrook 7H10 containing 10% Oleic Acid Albumin Dextrose Catalase (OADC) supplement was added to each well and homogenized using Multidrop Combi Reagent Dispenser (Thermo Fisher Scientific, Massachusetts, USA). All procedures were performed in a biosafety cabinet.

Bacterial cultures were grown in Middlebrook 7H9 broth complete at their preferred temperature, shaking at 180 rpm. Once cultures reached approximately OD600 of 1, they were aliquoted in sterile micro tubes, and frozen at –20 °C for future use. For the MIC_99_ determination, stock cultures were diluted to OD600 of 0.006 in Middlebrook 7H9 broth complete and 2 µL of the dilution were spotted into columns 1–11 of the 96-well plates. Plates were then incubated at the appropriate temperature and analyzed after confluent growth was observed in the growth control wells (column 11). Pictures of the plates were taken, and visual analysis was carried out to record the MIC_99_. At least three independent experiments were recorded for each species-antibiotic pair, for 15 antibiotics and 44 species, totalling 1980 individual MIC determinations.

### Sample preparation for metabolomics and proteomics

Sample preparation for LC-MS and proteomics was performed as previously described (Guzman et al., 2013). Mycobacterial species were grown in roller bottles in a volume of 100 mL of Middlebrook 7H9 broth complete to an OD600 of 1. Cultures were filtered using MF-Millipore Membrane Filter – 0.22 µm pore size to concentrate cell amount. For each species, 24 bacterial-laden filters were prepared and 3 were placed in petri-dish plates of Middlebrook 7H10 containing 10% OADC supplement and incubated at appropriate temperature (37 or 30 °C, depending on the species) for 5 doubling times, to expand the bacterial biomass. Subsequently, filters were transferred to fresh 7H10 containing 10% OADC supplement plates with vehicle or antibiotic at the concentration of 6×MIC99 and incubated for one doubling time, at appropriate temperature. Cells were then scraped into screw-cap tubes containing either 1 mL of ACN:MeOH:Water (2:2:1, v/v/v) and glass beads (150 μm) for LC-MS, or washed twice with 1 mL of PBS and then placed in 4% SDS/100 mM HEPES/50 mM DTT lysis buffer and glass beads (150–212 μm), for proteomics pilot experiment and 1% SDC/100 mM HEPES/50 mM DTT lysis buffer for remaining proteomics experiments. All samples were lysed by bead beating. At this stage, LC-MS samples were centrifuged, and the supernatant was collected and filtered using Corning Costar Spin-X Plastic Centrifuge 0.22 μm tube filters. Proteomics samples were heat killed and subjected to acetone precipitation, only when extracted with SDS, and peptide digested with LysC and Trypsin in 100 mM HEPES pH 8, Guanidine HCl 1 M.

### LC-MS metabolomics

LC-MS metabolomic samples were analyzed using a previously described method (Nandakumar et al., 2015). Briefly, aqueous normal phase liquid chromatography was performed using an Agilent 1200 LC system at where the samples were kept at controlled temperature (4 °C). A column Cogent Diamond Hydride Type C column linked to an Agilent Accurate Mass 6220 Time of Flight (TOF) spectrometer and coupled with an Agilent 1200 LC system was used for data acquisition. Flow rate of 0.4 ml min−1 was used and the elution of polar compounds was performed using a gradient of two solvents over a 24-min separation, A (MS-grade water and 0.1% formic acid) and B (acetonitrile and 0.1% formic acid) in positive mode. The gradient of solvent B was defined as 0–2 min 85%, 3–5 min 80%, 6–7 min 70%, 8–9 min 70%, 10–11 min 50%, 11–14 min 20%, 14–24 min 5% followed by a re-equilibration of 10 min at 85%. Using an isocratic pump, a reference mass solution was infused with the run to allow for simultaneous mass axis calibration. The data were analyzed by MassHunter Qualitative Analysis B07.00 or XCMS (Serafini et al., 2019). To verify the intracellular drug concentration for all samples, the molecular formulae of each drug was searched against the raw spectra and the integrated EIC was used to extract quantitative information. The peak height of both the antibiotic standard and the samples was used to calculate the relative drug concentration in each sample. Although area would generally be the variable of choice for performing quantifications, the peak shape was not consistent for LZD, therefore the peak height would correlate better with the amount of drug inside the cell. Further, the amount of drug added to the cells was considered and used to perform normalization, given that each species was exposed to a concentration of drug proportional to their MIC_99_. For identifying and quantifying Ribosyl-RIF, XCMS was used to perform peak picking and alignment. Metaboanalyst was used to perform batch effect corrections and statistical analysis (Smith et al., 2006). A feature with m/z 955.4601 was identified at 5.8 min and confirmed to be only present in RIF-treated samples. The feature abundance was normalized by the abundance in the pooled biological quality control (PBQC) samples (shown in Supplementary file 5). This accurate mass was searched using MassHunter Qualitative Analysis B07.00 with an acceptable error of ±10 ppm and the obtained peaks were integrated to extract the peak area, which was normalized by the peak area of the PBQC (shown in Figure 4). Finally, the average and the standard deviation between normalized values from six biological replicates were calculated and plotted.

### Proteomics

Proteomics analyses were performed at the Proteomics Scientific Technology Platform (STP) at The Francis Crick Institute. Data-dependent acquisition (DDA) was used to build a peptide library and Data-independent acquisition (DIA) was used to analyze the experimental samples. For both DDA and DIA, Evosep LC system (Evosep) (Krieger et al., 2019) was employed with the standard gradient for a total LC runtime of 44 min, using their supplied 15 cm column (Pang et al., 2021). Each sample was loaded from the peptide digests at a minimum volume of 10 µL (samples diluted for the optimum load for final loading volume of 10 µL to ensure all liquid enters the tip). An aliquot of the recommended amount of iRT peptides (Biognosys AG, Switzerland) was added to each sample at the sample loading stage. The protocol supplied with the Evotips was followed for conditioning/equilibrating/loading and washing the tips.

The outlet of the analytical column was connected directly to an adapter that allowed the EasySpray nano-source to be employed on the Orbitrap Fusion Lumos (Thermo Fischer Scientific, USA) using a stainless-steel emitter. The spray voltage was set to 2.2 kV. The default charge state was set to 2+. For the DDA runs, MS1 data were acquired in profile mode at a resolution of 60,000 (FWHM), with an AGC target of 1E6 ions and a maximum injection time of 50 ms. The ion funnel RF was set at 30%. Quadrupole isolation was employed over the MS1 mass range of 375–1200 m/z. The monoisotopic precursor selection (MIPS) was set to ‘Peptide’ and an intensity threshold of 5E4 was applied. Charge states from 2+ to 6+ were considered for MS/MS and dynamic exclusion was set to 15 s/10 ppm including exclusion of isotopes. Cycle time for the Data Dependent MS/MS Acquisition was set to 1 s. For the MS/MS, quadrupole isolation was set to 1.4 Da and HCD collision energy was employed at 32%. Data were acquired in the Orbitrap at a resolution of 15000 (FWHM) in centroid, with a fixed first mass of 120 m/z. The AGC was set at 1E6 and maximum injection time of 22 ms.

For the DIA, the following parameters were adjusted. The default charge state was set to 4+, and MS1 data were acquired in profile mode at a resolution of 120,000 (FWHM) with an AGC setting of 1E6 and maximum injection time of 20ms. The MS1 scan range was set from 393 to 907 m/z to allow enough data points per peak. 27 DIA windows (20 Da / 1 Da overlap) were employed over this range for DIA MS2 acquisition. These data were acquired at 30,000 resolution (FWHM) in the Orbitrap in centroid mode. HCD collision energy was the same as for the DDA runs. MS2 data were acquired over the mass range 200–2000 m/z with an AGC setting of 1E6 and a maximum injection time of 54 ms. Ions were injected for all available parallelizable time.

### Gene knockdown using CRISPRi

Gene silencing of *M. conceptionense arr*-1 and *arr*-X and both genes was performed following the protocols for *M. smegmatis* described in Wong and Rock, 2021 with the primers listed in Supplementary file 4.

Electrocompetent *M. conceptionense* cells were transformed with one of the three silencing constructs and with the empty vector pLJR962 as negative control. Transformants were selected by plating cells into Middlebrook 7H9 broth complete and 20 µg/mL of kanamycin.

Individual colonies were picked and inoculated into 10 mL of Middlebrook 7H9 broth complete and 20 µg/mL of kanamycin and glycerol stocks were prepared for subsequent experiments. To test for antibiotic sensitivity of knockdown constructs, glycerol stocks were used to inoculate 5 mL of Middlebrook 7H9 broth complete with 20 µg/mL of kanamycin and incubated at 37 °C with shaking to an OD600 of ca. 1. These saturated cultures were then used to inoculate fresh 5 mL aliquots of medium with kanamycin to an OD600 of 0.05 and the cultures were incubated at 37 °C with shaking to an OD600 of 0.4–0.8. The cultures were then diluted to an OD 0.1 in Middlebrook 7H9 broth complete with 20 µg/mL of kanamycin and 100 µg/mL of anhydrotetracycline (ATc) and grown at 37 °C with shaking to an OD600 of 0.4–0.8. This step was repeated a second time. Cultures were then streaked into Middlebrook 7H10 plates containing 10% OADC, 0.05% glycerol, 20 µg/mL of kanamycin, 200 µg/mL of ATc and rifabutin at 0.5 x, 1 x and 2 x the MIC99.

### Cloning, expression, and purification of Arr enzymes

The RIF ADP-ribosyl transferase (arr) genes from *M. smegmatis*, *M. flavescens,* and *M. conceptionense* were cloned from genomic DNA by PCR and inserted via isothermal assembly into the pNIC-CTHF expression vector (a gift from Opher Gileadi; Addgene plasmid #26105; Wong and Rock, 2021). The resulting plasmids were Sanger sequenced to confirm the correct insertion of the genes and then transformed into *E. coli* BL21(DE3) Gold competent cells (Agilent Technologies).

The transformed cells with each of the arr genes were grown at 37 °C and 200 rpm in 1 L of lysogeny broth (LB) supplemented with 50 µg/mL kanamycin to an OD600 of 0.6, at this point the temperature was dropped to 16 °C. Protein expression was induced by addition of isopropyl β-D-thiogalactopyranoside (IPTG) to a final concentration of 0.5 mM, and cells allowed to grow for 20 hr. Cells were harvested by centrifugation at 4000 × *g* for 30 min and stored at –80 °C.

The purification was performed according to the previously reported protocols (Koteva et al., 2018). In summary, cells were thawed on ice for 30 min before being resuspended in buffer A (50 mM HEPES (pH 7.5), 1 mM EDTA) containing a tablet of cOmplete EDTA-free protease inhibitor cocktail (Roche), 2 µL Benzonase nuclease (Millipore), and 6 mM MgCl2. Samples were further lysed by probe sonication (amplitude 35%, on 10 s, off 50 s, 2 min total on time per cycle, 2–3×cycles) and centrifuged at 48,000 × *g* for 45 min to separate the cell debris. The supernatant was filtered through a 0.45 µm membrane and loaded onto a 20 mL HiPrep Q Sepharose Column (GE Healthcare), pre-equilibrated with buffer A. The column was washed with five column volumes (CV) of 5% buffer B (50 mM HEPES (pH 7.5), 1 mM EDTA, 1 M NaCl), and the adsorbed proteins were eluted with five CV of a linear gradient from 10% to 30% buffer B. Fractions containing the desired Arr protein were pooled together and brought to 1.25 M (NH4)2SO4 by dropwise addition of the ammonium sulfate solution while stirring. After 30 min, the sample was filtered through a 0.45 µm membrane and loaded onto a 1 mL HiTrap Phenyl Sepharose column (GE Healthcare) pre-equilibrated with buffer C (50 mM sodium phosphate (pH 7.0), 1.25 M (NH4)2SO4). The column was washed with 15 CVs of 10% buffer D (50 mM sodium phosphate [pH 7]), and the adsorbed proteins were eluted with 20 CV of a linear gradient from 10 to 90% buffer D. Fractions were analyzed by sodium dodecyl sulphate−polyacrylamide gel electrophoresis (SDS−PAGE; NuPAGE Bis-Tris 4–12% Precast gels, Thermo Fisher Scientific), pooled, dialyzed against 2x2 L of 20 mM HEPES (pH 8.0), concentrated using 5000-molecular-weight-cut-off (MWCO) centrifugal ultrafiltration membranes (Millipore), aliquoted, and stored at –80 °C. The concentration was determined spectrophotometrically (NanoDrop, Thermo Fisher Scientific) at 280 nm using a theoretical extinction coefficient of 16960 M-1 cm-1 (ExPASy’s ProtParam; Savitsky et al., 2010).

### In vitro activity assay of Arr enzymes

Assays were carried out based on the previously reported methods (Koteva et al., 2018). Briefly, 150 nM enzyme (except for *M. conceptionense* Arr-X where 50 nM was used) in 50 mM HEPES buffer (pH 7.5) was mixed with 150 μM of RIF and 2 mM NAD^+^. Time points (50 μL) were quenched by the addition of methanol (200 μL). The samples were then analyzed by injecting 20 µL onto the HPLC column Poroshell 120 Å, EC-C18, 3.0 x 150 mm, 2.7 µm (Agilent Technologies) and monitoring the consumption of RIF and formation of ADP-ribosylated RIF product. The same protocol was followed for each of the rifamycins in the study: rifabutin, rifapentine, rifaximin, rifamycin B, and rifamycin S. A standard curve of each rifamycin was measured independently.

### Arr distribution analysis

In order to build a phylogenetic tree of mycobacterial Arr enzymes, Arr-ms (Uniprot A0QRS5) was used to query a local BLAST (Gasteiger, 2005; Eddy, 2009) database of genomic coding sequences of the mycobacterial reference genomes available in NCBI (Durão et al., 2018). The matching sequences were combined with those obtained by searching UniProt (Camacho et al., 2009) entries matching the RIF-ART protein family (Pfam PF12120 Consortium, 2022). CD-Hit (Savitsky et al., 2010; Li and Godzik, 2006) and Jalview (Waterhouse et al., 2009) were used to reduce redundancy (Castresana, 2000) and a multiple protein sequence alignment was calculated using MUSCLE (Edgar, 2004). Trees were generated with IQ-Tree 1.6.11 (Kalyaanamoorthy et al., 2017; Nguyen et al., 2015; Stamatakis, 2014) with 1000 ultrafast bootstrap replicates.

## Data Availability

Microbiological and biochemical data is available in the manuscript and supplementary information. Metabolomics and proteomics data are freely available in Zenodo (metabolomics - 19469446 and proteomics - 19627825). The following datasets were generated: Teixeira SubtilF Sorio de CarvalhoLP Garza-GarciaA SkehelM KirkpatrickJ DouglasH MachadoT 2026A macroevolution-inspired approach to reveal novel antibiotic resistance mechanisms - LC-MS MetabolomicsZenodo10.5281/zenodo.19469446PMC1324600042253228 Teixeira SubtilF Sorio de CarvalhoLP Garza-GarciaA SkehelM KirkpatrickJ DouglasH MachadoT 2026A macroevolution-inspired approach to reveal novel antibiotic resistance mechanisms - LC-MS ProteomicsZenodo10.5281/zenodo.19627825PMC1324600042253228

## Additional files

Supplementary file 1. Information on antibiotics used in this study.

Supplementary file 2. List of mycobacterial Arr orthologues.

Supplementary file 3. Primary sequence variation of Arr 1 and 3.

Supplementary file 4. MIC for different rifamycins and sgRNA sequences.

Supplementary file 5. Rifampin ADP-Ribosyltransferase phylogenetic tree calculation and sequence IDs.

MDAR checklist
