# Supplementary material for: A macroevolution-inspired approach to reveal novel antibiotic resistance mechanisms
Source: eLife. 2026 Jun 8;13:RP101940. doi: 10.7554/eLife.101940 (PMC13246000; doi:10.7554/eLife.101940)
Supplement: Peer review history

# Editor report: eLife Assessment

**DOI:** 10.7554/eLife.101940.3.sa0

**Authors:** Bavesh D Kana

**Affiliations:** University of the Witwatersrand South Africa

**Keywords:** Convincing, Important

This **important** study introduces an approach to discovering antibiotic resistance determinants by leveraging diverse susceptibility profiles among related mycobacterial species, with particular relevance to high-level resistance against natural product-derived antibiotics. The research provides **convincing** evidence for the role of ADP-ribosylation enzymes in rifamycin resistance among mycobacteria, whilst also demonstrating that antibiotic susceptibility is not correlated with growth rate or intracellular compound concentration. The revision is substantively improved, but some broader claims still require additional experimental support. This work lays a significant foundation for understanding the complexity of antibiotic resistance mechanisms in mycobacteria and opens new avenues for future antimicrobial research.

# Referee report: Reviewer #1 (Public review)

**DOI:** 10.7554/eLife.101940.3.sa1

This work analyzes innate resistance to drugs in mycobacteria by comparing minimum inhibitory concentrations (MICs) across a diverse panel of mycobacterial species. The results show that MICs are poorly correlated with growth rate while phylogeny associated with horizontal gene transfer underlies the observed differences in MIC, an important demonstration. A further investigation into the driver for the vast differences in susceptibility profiles shows that for three drugs the MIC is not correlated with intrabacterial drug concentrations where intrabacterial drug concentration is comprised of cytosolic and cell wall associated drug. This is a striking observation. The authors delve into the mechanisms that drive resistance to rifamycins and confirm that resistance is driven by ADP-ribosyltransferases of which two variant groups exist, one of which is kinetically faster and apparently is superior at modifying more hydrophobic rifamycins. The relative role of the two ADP-ribosyltransferases in conferring resistance especially in the species with both orthologs is not fully understood since the modified drug can possibly be further modified and transcriptional downregulation experiments performed in this work do not provide genetic evidence of perturbation of mRNA levels of the respective open reading frames.

Comments on revisions:

Demonstration of the level of transcriptional downregulation of the two Arr orthologs would have been a nice demonstration of (1) the utility of CRISPRi in other mycobacteria, (2) that the difference in rifabutin susceptibility during knockdown of Arr-1 vs Arr-X can fully be ascribed to the role of Arr-X in modifying the drug.

# Referee report: Reviewer #3 (Public review)

**DOI:** 10.7554/eLife.101940.3.sa2

This manuscript presents a macroevolutionary approach to identification of novel high-level antibiotic resistance determinants that takes advantage of the natural genetic diversity within a genus (mycobacteria, in this case) by comparing antibiotic resistance profiles across related bacterial species and then using computational, molecular, and cellular approaches to identify and characterize the distinguishing mechanisms of resistance. The approach is contrasted with "microevolutionary" approaches based on comparing resistant and susceptible strains of the same species and approaches based on ecological sampling that may not include clinically relevant pathogens or related species. The potential for new discoveries with the macroevolution-inspired approach is evident in the diversity of drug susceptibility profiles revealed amongst the selected mycobacterial species and the identification and characterization of a new group of rifamycin-modifying ADP-ribosyltransferase (Arr) orthologs of previously described mycobacterial Arr enzymes. Additional findings that intra-bacterial antibiotic accumulation does not always predict potency within this genus, that M. marinum is a better proxy for *M. tuberculosis* drug susceptibility than the commonly used saprophyte *M. smegmatis*, and that susceptibility to semi-synthetic antibiotic classes is generally less variable than susceptibility to antibiotics more directly derived from natural products strengthen the claim that the macroevolutionary lens is valuable for elucidating general principles of susceptibility within a genus.

There are some limitations to the work. The argument for the novelty of the approach could be better articulated. While the opportunities for new discoveries presented by identification of discrepant susceptibility results between related species is evident, it is less clear how the macroevolutionary approach is further leveraged for the discovery of truly novel resistance mechanisms. The example of the discovery of Arr-X enzymes presented here relied upon foundational knowledge of previously characterized Arr orthologs. There is less clarity about what the pipeline would look like for discovery of previously unknown determinants when one is agnostic to putative mechanisms. From the point at which interspecies differences in susceptibility are noted, does the framework still remain distinct from other discovery frameworks and approaches?

While the experimentation and analyses performed are generally well designed and rigorous, there are a few instances in which broad claims are based on inferences from sample sets or data sets that are, at present, too limited to provide robust support. For example, the claim that rifampicin modification, and precisely ADP-ribosylation, is the dominant mechanism of resistance to rifampicin in mycobacteria is still a bit premature or at least an over-generalization, as other enzymatic modification mechanisms and other mechanisms such as helR-mediated dissociation of rifampicin-stalled RNA polymerases, efflux, etc were not examined. CRISPR interference was used in a demonstrative example to support this assertion, but would need to be applied more systematically to be more conclusive. The general claim that intra-bacterial antibiotic accumulation does not predict potency in mycobacteria may be another over-generalization based on the limited set of drugs and species studied.

Comments on revisions:

Discussion, lines 321-323: "We found that resistance to these antibiotics in mycobacteria do not correlate with by uptake/efflux mechanisms in the species tested..." is an over-generalization and conflicts with the following statement on lines 199-201: "for BDQ we could observe some correlation between antibiotic potency and [BDQ]IB which could be indicative of efflux playing a role in antibiotic efficacy. Given that the current statement in the Discussion only applies to 2 of 3 drugs tested, a more specific or nuanced interpretation seems warranted.

# Author comment: Author response

**DOI:** 10.7554/eLife.101940.3.sa3

**Authors:** Fernanda T Subtil, Teresa FG Machado, Holly Douglas, Joanna M Kirkpatrick, Mark Skehel, Acely Garza-Garcia, Luiz Pedro S de Carvalho

**Affiliations:** The Francis Crick Institute London United Kingdom; The Francis Crick Institute London United Kingdom; The Francis Crick Institute London United Kingdom; The Francis Crick Institute London United Kingdom; The Francis Crick Institute London United Kingdom; The Francis Crick Institute London United Kingdom; UF Scripps Institute for Biomedical Innovation and Technology Jupiter United States

The following is the authors’ response to the original reviews

**Public Reviews:**

**Reviewer #1 (Public review):**

This work shows that resistance profiles to a variety of drugs are variable between different mycobacterial species and are not correlated with growth rate or intrabacterial compound concentration (at least for linezolid, bedaquiline, and Rifampicin). Note that intrabacterial compound concentration does not distinguish between cytosolic and periplasmic/cell wall-associated drugs. The susceptibility profiles for a wide range of mycobacteria tested under the same conditions against 15 commonly used antimycobacterial drugs provide the first recorded cross-species comparison which will be a valuable resource for the scientific community. To understand the reasons for the high Rifampicin resistance seen in many mycobacteria, the authors confirm the presence of the arr gene known to encode a Rif ribosyltransferase involved in Rif resistance in *M. smegmatis* in the resistant mycobacteria after confirming the absence of on-target mutations in the RpoB RRDR. Metabolomic analyses confirm the presence of ribosylated Rif in some of the naturally resistant mycobacteria which may not be entirely surprising but an important confirmation. Presumably M. branderi is highly resistant despite lacking the arr homolog due to the rpoB S45N mutation. *M. flavescens* has an MIC similar to that of *M. smegmatis*, despite having both Arr-1 and Arr-X. Various Arr-1 and Arr-X proteins are expressed and characterized for catalytic activity which shows that Arr-X is a faster enzyme,, especially with respect to more hydrophobic rifamycins. *M. flavescens* has similar MIC values to Rifapentine and Rifabutin to *M. smegmatis*. Thus, the Arr-1 versus Arr-X comparison does not provide a complete explanation for the underlying reasons driving natural Rif resistance in mycobacteria. Downregulation of Arr-X expression in M. conceptionense confers increased sensitivity to Rifabutin confirming its role as a rifamycin-inactivating enzyme.

Overall, the comparison of cross-species susceptibility profiles is novel; the demonstration that MIC is not correlated with intracellular drug concentration is important but not sufficiently interrogated, the demonstration that Arr-X is also a Rif ADP-ribosyltransferase is a good confirmation and shows that it is more efficient than Arr-1 on hydrophobic rifamycins is interesting but maybe not entirely surprising. The manuscript seems to have two parts that are related, but the rifamycin modification aspect of the work is not strongly linked to the first part since it interrogates the modification of one drug but not the common cause of natural resistance for other drugs.

**Reviewer #2 (Public review):**

Summary:

The authors use a variety of methods to investigate the mechanisms of innate drug resistance in mycobacteria. They end up focusing on two primary determinants - drug accumulation, which correlates rather poorly with resistance for many species, and, for the rifamycins, ADP-ribosyltransferases. The latter enzymes do appear to account for a good deal of resistance, though it is difficult to extrapolate quantitatively what their relative contributions are.

Overall, they make excellent use of biochemical methods to support their conclusions. Though they set out to draw very broad lessons, much of the focus ends up being on rifamycins. This is still a very interesting set of conclusions.

Strengths:

(1) A very interesting approach and set of questions.

(2) Outstanding technical approaches to measuring intracellular drug concentrations and chemical modification of rifamycins.

(3) Excellent characterization of variant rifamycin ADP-ribosyltransferases

Weaknesses:

(1) Figure 3c/d: These panels show the same experiment done twice, yet they display substantially different results in certain cases. For instance, *M. smegmatis* appears to show an order of magnitude lower RIF accumulation in panel d compared to M. flavescens, despite them displaying equal accumulation in panel c. The authors should provide justification for this variation, particularly as quantitative intra-species comparisons are central to the conclusions of this figure.

The data in panels 3c and 3d are from different sets of experiments. The reviewer is correct with regards to *M. smegmatis*. The data indeed is ~ 1 order of magnitude different. However, the data for other species is very similar. The reviewer may also have noticed that the error bars are also larger in 3d, compared to 3c, indicating a greater variation between independent experiments use in 3d. We do not have a good explanation for this, other than the experiments shown in 3d were associated with greater biological variability.

(2) There are several technical concerns with Figure 3 that affect how to interpret the work. According to the methods, the authors did not appear to normalize to an internal standard, only to an external antibiotic standard (which may account for some of the technical variation alluded to above).

We agree that using a labeled drug as an internal standard (IS) would be ideal. However, the experiment initially followed an untargeted metabolomics approach, which later shifted to relative drug quantification. At that stage, normalizing with IS was impractical because proper implementation would require multiple IS across the chromatographic range. Therefore, we opted for total ion current (TIC) normalization, which accounts for variability in overall metabolite abundance—even though the experimental setup was already adjusted for each bacterial species’ growth rate. Additionally, we prepared external standard curves for each drug to enable quantification, and the amount of drug added to each plate was considered when reporting these values.

Second, the authors used different concentrations of drug for each species to try to match the species' MICs. I appreciate the authors' thinking on this, but I think for an uptake experiment it would be more appropriate to treat with the same concentration of drug since uptake is likely saturable at higher drug concentrations. In the current setup, for the species with higher MIC, they have to be able to uptake substantially more antibiotics than the species with low MIC in order to end up with the same normalized uptake value in Figure 3d. It would be helpful to repeat this experiment with a single drug concentration in the media for all species and test whether that gives the same results seen here.

We respectfully disagree with the reviewer. Experiments such as the one proposed by the review work well when MIC values are a few fold apart, for strains of the same species, but have not been tested when MIC values are 100-1000-fold apart, with different species. Furthermore, what would be the interpretation of compound uptake at 1000-fold the MIC for one species and MIC level for another? By using antibiotic concentrations at the respective MIC for each species we are at least under conditions where we know the biological effect of the antibiotic across species is the same, based on its potency.

(3) Figure 4f: This panel seems to argue against the idea that the efficacy of RIF ribosylation is what's driving drug susceptibility. M. flavescens is similarly resistant to RIF as *M. smegmatis*, yet M. flavescens has dramatically lower riboslyation of RIF. This is perhaps not surprising, as the authors appropriately highlight the number of different rif-modifying enzymes that have been identified that likely also contribute to drug resistance. However, I do think this means that the authors can't make the claim that the resistance they observe is caused by rifamycin modification, so those claims in the text and figure legend should be altered unless the authors can provide further evidence to support them. This experiment also has results that are inconsistent with what appears to be an identical experiment performed in Supplemental Figure 5b. The authors should provide context for why these results differ.

In regard to enzyme efficiency, the apparent rate of all Arr-1 is relatively similar in converting RIF into ADP-Ribosyl-Rif between species. However, Arr-X is much more efficient when compared to Arr-1 in both *M. flavescents* and *M. conceptionense*. This is indicated by the apparent rate measured and displayed on figure 5c.

Proteomics data shows that there is upregulation of Arr-1 and Arr-X upon rifampicin treatment in *M. flavescens* and *M. conceptionense*. However, the same experiment was not performed in Arr-1 KD. Therefore, we can’t verify through this approach if the activity observed in vivo directly correlates with a higher expression of Arr-X alone. Of note, likely both enzymes contribute to resistance to rifamycins, as per our results with the Arr-X KD and sensitization of *M. conceptionense* to RIF.

**Author response image 1.** 

It is also worth mentioning that there are other enzymes in the pathway of RIF ribosylation and their efficiency is unknown (Author response image 2). Therefore ADP-Ribosyl-RIF It is not an “end-metabolite” and maybe not the sole determinant of RIF resistance via ADP-ribosylation. Downstream enzymes can also account for the difference observed between *M. flavescens* and *M. smegmatis*.

**Author response image 2.** 

It is correct that the Rifampicin MIC for *M. flavescens* is the same as *M. smegmatis*.

(4) Fig 4f/5c: M. flavescens has both Arr-1 and Arr-X, yet it appears to not have ribosylated RIF. This result seems to undermine the authors' reliance on the enzyme assay shown in Fig 5c - in that assay, M. flavescens Arr-X is very capable of modifying rifampicin, yet that doesn't appear to translate to the in vivo setting. This is of importance because the authors use this enzyme assay to argue that Arr-X is a fundamentally more powerful RIF resistance mechanism than Arr-1 and that it has specificity for rifabutin. However, the result in Figure 4f would argue that the enzyme assay results cannot be directly translated to in vivo contexts. For the authors to claim that Arr-X is most potent at modifying rifabutin, they could test their CRISPRi knockdowns of Arr-X and Arr-1 under treatment with each of the rifamycins they use in the enzyme assay. The authors mentioned that they didn't do this because all the strains are resistant to those compounds; however, if Arr-X is important for drug resistance, it would be reasonable to expect to see sensitization of the bacteria to those compounds upon knockdown.

The reviewer is reading Fig. 4f incorrectly, probably because it is plotted in a linear scale instead of logarithmic scale. Ribosylated Rif is present in *M. flavescens*, just at lower levels than *M. conceptionense* and *M. smegmatis*. In species where there is no Arr-1 or Arr-3, ribosylated RIF is not detected at all (e.g. *M. tuberculosis*), i.e., concentration is zero. Therefore, any detection of ribosylated RIF can be considered significant. In addition, as mentioned before, ADP-ribosylation of RIF is not the final product of the reaction and further studies need to be undertaken to understand subsequent reactions.

(5) Figure 5d: The authors use this CRISRPi experiment to claim that ArrX from M. conceptionanse is more potent at inactivating rifabutin than Arr-1. This claim depends on there being equal degrees of knockdown of Arr-1 and Arr-X, so the authors should validate the degree of knockdown they get. This is particularly important because, to my knowledge, nobody has used this system in M. conceptionanse before.

We agree with the reviewer that a qPCR should have been performed to define the extent of interference in the strain. generated Unfortunately, at this time a qPCR was not performed in the strains tested to confirm the extent of down regulation. Although it is the best practice to validate the strain KD, there is no indication that the effect observed is due to unspecific downregulation. The genetic environment in which Arr-X is positioned is different from Arr-1 and the targeting oligonucleotides are specific and would not promiscuously bind to Arr-1. Said that, this is indeed a fault in our setup.

(6) The authors' arguments about Arr-X and Arr-1 would be strengthened by showing by LC/MS that Arr-X knockdown in M. conceptionense results in more loss of ribosyl-rifabutin than knockdown of Arr-1.

We agree with the reviewer that performing the LC-MS analysis of the Arr-x knockdown would have strengthened the argument of our paper. Unfortunately, this experiment was not performed.

**Reviewer #3 (Public review):**

This manuscript presents a macroevolutionary approach to the identification of novel high-level antibiotic resistance determinants that takes advantage of the natural genetic diversity within a genus (mycobacteria, in this case) by comparing antibiotic resistance profiles across related bacterial species and then using computational, molecular, and cellular approaches to identify and characterize the distinguishing mechanisms of resistance. The approach is contrasted with "microevolutionary" approaches based on comparing resistant and susceptible strains of the same species and approaches based on ecological sampling that may not include clinically relevant pathogens or related species. The potential for new discoveries with the macroevolution-inspired approach is evident in the diversity of drug susceptibility profiles revealed amongst the selected mycobacterial species and the identification and characterization of a new group of rifamycin-modifying ADP-ribosyltransferase (Arr) orthologs of previously described mycobacterial Arr enzymes. Additional findings that intra-bacterial antibiotic accumulation does not always predict potency within this genus, that *M. marinum* is a better proxy for *M. tuberculosis* drug susceptibility than the commonly used saprophyte *M. smegmatis*, and that susceptibility to semi-synthetic antibiotic classes is generally less variable than susceptibility to antibiotics more directly derived from natural products strengthen the claim that the macroevolutionary lens is valuable for elucidating general principles of susceptibility within a genus.

There are some limitations to the work. The argument for the novelty of the approach could be better articulated. While the opportunities for new discoveries presented by the identification of discrepant susceptibility results between related species are evident, it is less clear how the macroevolutionary approach is further leveraged for the discovery of truly novel resistance determinants. The example of the discovery of Arr-X enzymes presented here relied upon foundational knowledge of previously characterized Arr orthologs. There is little clarity on what the pipeline for identifying more novel resistance determinants would look like. In other words, what does the macroevolutionary perspective contribute to discovery from the point of finding interspecies differences in susceptibility? Does the framework still remain distinct from other discovery frameworks and approaches? If so, how?

Thanks for pointing this out, as this is a critical feature of our study and method. Our approach relies on inter-species comparative genomics and phenotypes, and therefore, it is distinct from inter-strains comparison. This difference is dramatic, and it becomes clearer when we are comparing the core genome of *M. tuberculosis* (one species) 92% with the core genome of the genus, circa of 1%. While we focus on rifamycin in this manuscript, future manuscripts will investigate many of the other dozens of “inconsistencies” observed between the genetic makeup of different mycobacterial species and there actual performance in the presence of different antibiotics.

While the experimentation and analyses performed appear well-designed and rigorous, there are a few instances in which broad claims are based on inferences from sample sets or data sets that are too limited to provide robust support. For example, the claim that rifampicin modification, and precisely ADP-ribosylation, is the dominant mechanism of resistance to rifampicin in mycobacteria may be a bit premature or an over-generalization, as other enzymatic modification mechanisms and other mechanisms such as helR-mediated dissociation of rifampicin-stalled RNA polymerases, efflux, etc were not examined nor were CRISPRi knockdown experiments conducted beyond an experiment to tease out the role of Arr-X and Arr-1 in one strain. The general claim that intra-bacterial antibiotic accumulation does not predict potency in mycobacteria may be another over-generalization based on the limited number of drugs and species studied, but perhaps the intended assertion was that antibiotic accumulation ALONE does not predict potency.

**Recommendations for the authors:**

**Reviewer #1 (Recommendations for the authors):**

Major comments

(1) The metabolomics is done using mycobacteria grown on filters. Initially, mycobacterial cells are grown on the filters for 5 doublings before being transferred to drug-containing (or free) agar for one doubling. Is this based on calculated doubling time in liquid culture or a true determination of the fact that the biomass increases to what would amount to 5 doublings?

The doubling time used is the one determined in liquid media. Although it is possible that the growth kinetics in solid media is slightly different from liquid (±10%), this experimental design is well established for *M. tuberculosis* (since Proc Natl Acad Sci U S A. 2010 May 25;107(21):9819-24.) and *M. smegmatis* (unpublished). Therefore, we used the growth rate as a proxy for having the same biomass of cells for each species tested. A maximum difference of 10% was observed between *M. tuberculosis* growth in liquid and in solid media, however, cells grow exponentially for much longer in filters. This makes filter-based experiments more reliable, as few growth phase-derived differences are present.

(2) The demonstration that intrabacterial drug concentrations vary between mycobacterial species in a manner not related to MIC for at least LZD and RIF, is an important finding. However, intrabacterial does not mean cytoplasmic since a considerable fraction could be present in the periplasmic/cell wall layers. Ideally, this would need to be determined but would of course be a massive undertaking since the method needs validation & optimization for each mycobacterial species. Nevertheless, this has to be mentioned. In addition, three drugs are limiting. Measuring additional drug concentrations in these 5 mycobacteria would at least establish some confirmation about the extent of this lack of correlation. Thus, could the authors measure concentrations of additional drugs with intracellular targets?

Testing additional drugs can be beneficial and would be an expansion of our paper, which will definitely be on future plans for further studies focusing on other antibiotics described here. It would also provide new insights into other possible mechanisms of resistance in mycobacterial species. However, in this study we aimed to first determine the antibiotic response profile in different mycobacterial species, and once we identified interesting resistance phenotypes that could not be readily explained by known mechanisms of resistance, we narrowed it down to certain drugs and species that would potentially provide insights into new mechanisms of antibiotic resistance. Finally, exploring drug concentration across multiple bacterial compartments is a dauting task and it has not been done extensively with any species, not to mention with multiple species, many of which are still lacking any study of their actual cell envelope.

(3) CRISPRi was used to reduce transcription in *M. conceptionense*. What was the level of gene downregulation?

As mentioned previously, a setback from our setup is that the level of KD was not measured at this instance.

Minor comments:

(1) The introduction mentions the fast and slow-growing mycobacteria which are classified based on the time that it takes to observe colonies on solid agar. However, in liquid medium, there is less correlation between the reported growth on agar and doubling time in liquid (Figure 1b, Figure 2d). This could be mentioned in the results section. In Figure 2d, the filled circles represent fast-growers but this does not hold well for liquid culture and it might make more sense to not distinguish between fast- and slow-growers in these graphs. A small complication would also be the fact that the doubling time represents growth in a liquid medium with Tyloxapol as a detergent whereas the MIC and metabolomics are done on solid agar with no detergent. The metabolomics is done after a doubling but for those where agar growth and liquid growth have large discrepancies in growth rate, there could be some differences.

Apologies for this misunderstanding. Fast- and slow-growth phenotypes are determined in Lowenstein-Jensen (LJ) agar, not in 7H10 agar (used in our study and most studies of mycobacteria). Furthermore, this is a qualitative definition, not a quantitative one. Therefore, our measurements do not need to correlate with fast- and slow-growth phenotypes, unless we had used that one specific medium. Furthermore, in liquid medium, we determined growth rate directly, which is never done with LJ medium.

In addition to adding the same amount of cells to each filter, we also perform TIC normalization, which should account for how rich the samples were – and therefore how much material we had. Therefore, we do not observe discrepancies due to differences in growth rate and the presence/absence of detergent in the media.

It is also worth mentioning that this experimental set up has been well established in many *M. tuberculosis* labs that study metabolism. Importantly, the use of detergent drastically affects mass spectrometry, and therefore cannot be used.

(2) Figure 1g in the text should be Figure 1f.

Apologies, it has been fixed.

(3) Figure S1 would be ideal to have in (supplementary) table format.

This data is now being provided in a table format.

(4) Table S1 - ethambutol misspelt.

Spelling has been corrected.

(5) MIC for species such as *M. abscessus* could depend on medium (7H9-based medium can give different MIC values than CAMH).

Indeed, different media can significantly change MIC values, and this is true for many bacterial species, if not all. For this study we used only species that could be grown in 7H9 broth containing 10 % ADC, 0.05% glycerol 0.05% tyloxapol and 7H10 plates containing 10% OADC and 0.05% glycerol. MIC_99_ was determined in the latter as we found more efficient and robust to do our tests it in solid media. The goal of our experiment was not to the determined the “true” MIC for the antibiotics tested, as this value does not exist. It was to find lack of correlations between relative values and the presence of genes that can account for it.

(6) The statement "the experiment was performed at a concentration of antibiotic equal to its MIC" initially seems confusing. It was not equal to the MIC but performed at 6-fold the respective MIC of the species in question. Maybe re-phrasing this would help.

Apologies for this oversight. It has been corrected.

(7) Note that some mutations outside the RRDR (eg. V170F and I491F) can also cause Rif resistance.

**Author response image 3.** Structure and sequence conservation of RpoB in selected mycobacterial species. (**A**) Rainbow diagram of RpoB X-Ray structure coloured according to sequence conservation. Dark purple indicates high conservation, whereas dark orange indicates low conservation. RIF (showed in magenta) is bound to RpoB. Zoomed view displays that the RIF-binding pocket is considerably conserved. (**B**) RpoB protein sequence has an 81bp region called Rifampicin Resistance Determining Region (RRDR) that is known to be important for RIF binding and is where most mutations occur in drug-resistant TB. Sequence alignment displays that the RRDR region is conserved with the exception of *M. branderi*, which has an Asn instead of a Ser residue in position 456 (numbering is related to the *M. tuberculosis* sequence), highlighted in bold.

Attached we have a structural alignment of RpoB of the species highlighted on this paper. Although there is variability within the sequences, which is also displayed in Author response image 3 with the conservation analysis, the residues that have been implicated with resistance (including V170 and I491) are conserved. Alignment sent on .fasta file that can be opened in jalview.

(8) Discuss how the RpoB S450N mutation in *M. branderi* confers the observed level of resistance.

That’s a great point, thank you. Now it reads as:

“The rifampicin (RIF) binding pocket is generally conserved, but *Mycobacterium branderi* has an S450N mutation in the RRDR region. While this specific mutation hasn't been found in clinical isolates, it's located at the binding site and may confer resistance (273). Although both serine (S) and asparagine (N) have similar side chains, related mutations like S450Q have been linked to resistance (156). Thus, M. branderi may be RIF-resistant due to this mutation. In contrast, *M. conceptionense*, *M. flavescens*, and *M. smegmatis* show no target sequence differences that explain their resistance”

(9) The statement that the three tested NTM are sensitive to rifabutin ("resistant to all rifamycins except for rifabutin") needs to be interpreted considering what sensitivity means. The MIC is still high (1.6-3.1 ug/mL) when compared to that of Mtb. The 2-fold differences in MIC between *M. smegmatis* and *M. conceptionense* do not really prove or disprove the role of Arr-X in rifabutin resistance.

We fixed the sentence to be more careful with the language on the text. We agree, but it is worth mentioning that generally with bacteria there is a regulation by the CLSI. Each bacterial species has a range that is considered sensitive or resistant, but these are not available for the species used in this study. In general, bacteria with MIC values above 8 µg/mL are considered resistant to rifampin (J Antibiot 2014 67:625).

(10) Figure 1d: It's hard to quantify the sensitivity of the plates. Can this be done by MIC? Was only rifabutin tested or also rifampicin?

The initial experiments described on the paper were all performed using Rifampicin only. Then, the MIC for the remaining rifamycins was determined for *M. smegmatis*, *M. flavescens* and *M. conceptionense*, and can be perused on “Supplementary table 4”. Figure 5d is to illustrate the effect of the KD in *M. conceptionense* sensitivity to rifabutin.

(11) Is there data to show the ADP-ribosylation of rifabutin in M. conceptionense and the CRISPRi strains?

Unfortunately, we did not perform LC-MS analysis on *M. conceptionense* CRISPRi strains exposed to rifabutin to measure potential ADP-ribosylation.

**Reviewer #2 (Recommendations for the authors):**

(1) It would be useful if the authors would complete Figure 1A by determining growth rates for the remaining 18 strains that they currently omitted.

These growth rates were obtained using roller bottles and in at least 3 independent experiments, unfortunately the throughput is far ideal. The goal of the experiment was to highlight difference in growth rate, beyond fast- and slow-growth, which we did. Adding the remaining values would not change this conclusion. Growth rate variation in 7H9 is significant and the point is made in our figure.

(2) The authors should justify their choice of species used in Figures 3-4. It would be useful to know, for instance, if the authors chose these species in an unbiased fashion, or if they were chosen because the authors had already determined that they possess rifamycin-modifying enzymes of interest. In that case, they wouldn't necessarily be a representative sample to use for the correlation analysis of antibiotic uptake and potency in Figure 3.

They were chosen because of their resistance profile for BDQ, LZD and RIF. This has been addressed in the text, which now reads “Given the antibiotic response profiles observed, we selected BDQ, LZD and RIF to explore the molecular causes of these dramatic changes in antibiotic potency observed across the Mycobacterium genus.”

(3) Figure 4b: The data in this panel appear inconsistent - for instance, M. houstonense appears to grow at 10X Mtb MIC, but fails to grow at 1X Mtb MIC. Repeating this experiment would better establish the validity of the authors' claims about the relative susceptibility of these strains to RIF.

The figures got rotated when exported from illustrator. Corrected figure is uploaded, and original plate photos are also uploaded for clarity.

(4) Figure 4e: Does Arr-X get upregulated in these proteomic datasets? The authors' argument that proteomic upregulation correlates with important drug resistance genes would imply that it might be, so that would be useful information to provide.

Arr-X is slightly upregulated, but not statistically significant – this could be due to the native expression of Arr-1. Data is displayed in a previous answer.

(5) I wasn't able to find the supplementary tables that the authors allude to - not sure if that was a file mixup, but those tables would be useful for interpreting the manuscript.

We are sorry that you couldn’t access the table. It must be a file corruption issues, as the other reviewers were able to. We will make sure that all tables are available and accessible.

(6) For LC/MS, the authors use peak height instead of peak area, which they argue correlates better with the amount of drug in cells because of the poor peak shape they observed for linezolid. This is not standard practice, so the authors should provide evidence to support this claim by running an LC/MS standard curve, then showing the correlation between peak height and amount of compound added as well as the correlation between peak area and compound.

Thank you for pointing that out, accuracy calculated and displayed. Both peak area and height can be used, but indeed area is standard practice.

(7) The authors should provide methods information about the LC column and the gradient settings used for LC-MS, as well as the settings of the MS.

The full method has been added to the paper.

**Reviewer #3 (Recommendations for the authors):**

I have only minor comments aside from the information in the Public Review:

(1) Results, section on Intra-bacterial antibiotic accumulation, line 8: "experiment was performed at a concentration of antibiotic PROPORTIONAL to its MIC" would be more accurate?

Agreed and adjusted according to Reviewer’s suggestion.

(2) Results, section on A minor role for pre-existing target modification, last sentence: the mere presence of RIF-ribosylating enzymes does not, in and of itself indicate that "RIF modification, and precisely ADP-ribosylation, is the dominant mechanism of resistance to RIF in mycobacteria", as other mechanisms and other forms of modifying enzymes are known to confer rifamycin resistance, with redundancy (e.g., other rifampicin-modifying enzymes, or helR-mediated dissociation of rifampicin-stalled RNA polymerases from DNA). It would be more appropriate to suggest the results presented to this point indicate RIF modification is common among mycobacteria. The evidence from the CRISPRi knockdown of Arrs shown in Fig 5d is the kind of evidence that suggests ribosylation as a dominant mechanism, at least against rifabutin in this particular species.

Absolutely, there are other possible modifying enzymes that could be encoded by these mycobacterial species. There is a possibility that *M. flavescens* and *M. smegmatis* encode for a putative *helR* (attached alignment) but further experiments would need to be carried out to confirm its ability to displace RIF in the RNAP. Interestingly, the presence of both Arr and HelR has been studied in *M. abscessus* and those mechanisms of resistance are independent from each other (Molecular Cell 2022 82(17):3166-3177.e5).

(3) Discussion, 2nd sentence needs grammatical editing.

Rephrased and it reads “Using our mycobacterial library, we identified for the first time high- and ultra-high-level intrinsic resistance (3) to many of the antibiotics tested. Of note, the resistant phenotype is naturally occurring and not a result of mutations due to exposure to the antibiotic in the clinic – which is the more traditional approach for probing mechanisms of antibiotic resistance. Our observations revealed that resistance profiles are highly variable across the genus and do not follow phylogeny, implicating HGT as the key mechanism for acquisition of resistance determinants and evolution of antibiotic resistance in mycobacteria (42).”

(4) Discussion, page 7, first line: the inclusion of LZD and BDQ in this statement seems at odds with Figure 2c and the statements in the first paragraph of page 5 highlighting these as examples of drugs to which most mycobacteria are susceptible.

Indeed, many of the species are susceptible, however the MIC_99_ levels observed have never been reported before, and therefore we found it to be an interesting finding to highlight. From a treatment perspective, knowing which species are sensitive to which drugs is of course the most useful outcome of our study.

(5) The next sentence..."We found that resistance to these antibiotics in mycobacteria cannot be explained by uptake/efflux mechanisms..." is a bit of an over-generalization and conflicts with the evidence presented earlier that efflux could be playing a role in BDQ resistance and the published evidence establishing a clinically significant role for efflux-mediated BDQ resistance in *M. tuberculosis*, *M. avium* complex and *M. abscessus* complex.

We rephrased it to make it more specific to our findings. It reads “We found that resistance to these antibiotics in mycobacteria do not correlate with by uptake/efflux mechanisms in the species tested and it does not correlate with growth rate. Identification of mycobacterial species highly resistant to BDQ and LZD is worrisome as most of this species, if not all, have never been exposed to these drugs.”

(6) Methods, section on In vitro activity assay of Arr enzymes, line 1: reference(s) should be provided for previously reported methods.

Reference now added.

(7) Figure 2d: the low end of the susceptibility range is not well defined.

In this figure the susceptibility is not defined as the lowest area of the graph, but the lower concentrations are indeed harder to be defined. Hopefully supplementary figure 1 and the additional table containing the MIC can be informative to address this comment.

(8) Figures 3c,d: the presentation of the relative antibiotic concentrations could be harmonized between the graphs in 3c and those in 3d to enable a more ready comparison.

We disagree. The goal of these different panels is exactly to illustrate two distinct points. C gives the relative concentration of antibiotic, while D correlates relative concentration with MIC99. The use of log scale in D further clarifies that there is no correlation between intracellular antibiotic concentration and potency (MIC). This information is not present in C.

(9) Figure 4f and Supplementary Figure 5b: it is difficult to understand the limited amount of ribsosyl-RIF in *M. flavescens* in Fig 4f relative to Supplementary Figure 5b (esp. when considering M. smeg as a common comparator); and, further, to understand the seeming lack of correlation between RIF susceptibility, ribosylation and Arr number and catalytic efficiency for these two strains without considering additional resistance mechanisms.

In reality the difference between figure 4f and Supplementary figure 5b is mainly due to *M. smegmatis* – that has an apparent lower production of ribosyl-RIF in the experiment described in the supplementary figure. The values for *M. flavescens* are relatively similar. In addition, the ADP-Ribosyl-RIF is not the final metabolite of the pathway.

In regards of having the entire picture, it is true that we were unable to completely unravel and correlate MIC value, expression of Arr-1, expression of Arr-3, efficiency of each enzyme, production of ADP-Ribosyl-RIF and the presence of other possible mechanisms of resistance and this is indeed a setback in our study, and of most studies ever published, which usually focus on one resistant determinant.
